# Supplementary material for: Four distinct network patterns of supramolecular/polymer composite hydrogels controlled by formation kinetics and interfiber interactions
Source: Nat Commun. 2023 Mar 27;14:1696. doi: 10.1038/s41467-023-37412-0 (PMC10042874; doi:10.1038/s41467-023-37412-0)
Supplement: Supplementary file 1 — Supplementary Information [file 41467_2023_37412_MOESM1_ESM.pdf]

Supplementary Information

Four distinct network patterns of supramolecular/polymer composite hydrogels controlled by formation kinetics and interfiber interactions

Keisuke Nakamura[1], Ryou Kubota[1]*, Takuma Aoyama[2,3], Kenji Urayama[3], Itaru Hamachi[1,4]*

[1]Department of Synthetic Chemistry and Biological Chemistry, Graduate School of Engineering, Kyoto University, Nishikyo-ku, Katsura, Kyoto 615-8510, Japan

[2]Department of Macromolecular Science and Engineering, Kyoto Institute of Technology, Matsugasaki, Kyoto 606-8585, Japan

[3]Department of Material Chemistry, Graduate School of Engineering, Kyoto University, Nishikyo-ku, Katsura, Kyoto 615-8510, Japan

[4]JST-ERATO, Hamachi Innovative Molecular Technology for Neuroscience, Nishikyo-ku, Katsura, Kyoto 615-8530, Japan

Correspondence:

rkubota@sbchem.kyoto-u.ac.jp

ihamachi@sbchem.kyoto-u.ac.jp

Table of contents

1. Supplementary methods

2. Supplementary figures

3. Supplementary tables

4. Captions for supplementary movies

5. Supplementary references

Supplementary methods

General. Unless stated otherwise, all commercial reagents were used as received. MES (2-(*N*-morpholino)ethanesulfonic acid (>99%)) and agarose were purchased from FUJIFILM Wako Pure Chemical Co. (product number: 341-01622) and nacalai tesque (product number: 01132-34), respectively. Thin layer chromatography (TLC) was performed on silica gel 60F254 (Merck). 1H NMR spectra were obtained on a Varian Mercury 400 and a JEOL JNM-ECZ500, and JMM-ECA600 spectrometer with tetramethylsilane (0 ppm) as the internal references. Reversed-phase HPLC (RP-HPLC) was carried out on a Hitachi Chromaster system equipped with a diode array and YMC-Pack Triart C18 or ODS-A columns. All commercially available reagents were used as received. BPmoc-F_3[1], NPmoc-F(F)F[2], Ald-F(F)F[3], GalNAc-cycC_6[4], Lys-cycC_5[5], Phos-MecycC_5[6], DBS-COOH[7], TMR-Gua[8], Alx546-cycC_6[9], and Alx488-Agarose[10] were synthesized as previously reported (see synthesis procedures). The images of confocal laser scanning microscopy (CLSM) were acquired by LSM 800 equipped with an Airyscan unit and a motorized stage (Carl Zeiss). A Fluar 5× objective lens (0.25 numerical aperture, Carl Zeiss) and a Plan-Apochromat 63× objective lens (1.40 numerical aperture, oil immersion, Carl Zeiss) were used for the low- and high-magnification imaging, respectively. High-resolution Airyscan images and movies were reconstructed from multiple images using the tiles tool of ZEN software (Carl Zeiss). 3D images were made by Imaris 9.5 (Bitplane). Pearson's correlation coefficients were calculated by Coloc 2 program in Fiji.[11,12] Microneedle stamps

were fabricated by microArch®S140 (Boston Micro Fabrication). Photographs were taken by iPhone 11 Pro (Apple).

Synthesis procedures.

Synthesis of BPmoc-F$_3$, NPmoc-F(F)F, and Ald-F(F)F

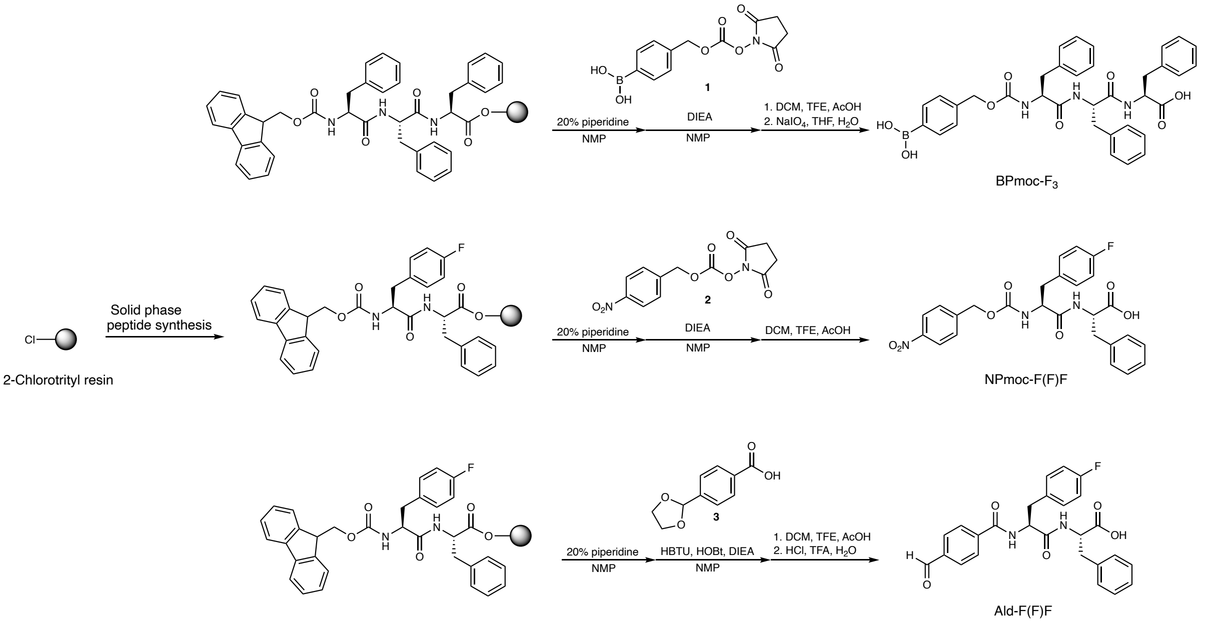

BPmoc-F$_3$, **NPmoc-F(F)F**, and **Ald-F(F)F** were prepared by Fmoc solid-phase peptide synthesis using a commercially available 2-chlorotrityl resin. The condensation reaction was carried out in the presence of Fmoc-protected amino acids (3.0 eq), 2-(1H-benzotriazol-1-yl)-1,1,3,3-tetramethyluronium hexafluorophosphate (HBTU: 3.0 eq), 1-hydroxybenzotriazole hydrate (HOBt·H_2O: 3.0 eq) and diisopropylethylamine (DIEA: 6.0 eq) in 1-methyl-2-pyrrolidinone (NMP). Removal of Fmoc protecting group was performed using NMP solution containing 20% piperidine. After removal of the Fmoc group on the terminal amino group, the resulting free amino group was allowed to react with BPmoc-OSu (**1**)[2] or NPmoc-OSu (**2**)[2] in the presence of DIEA (3.0 eq). For the synthesis of Ald-F(F)F, 4-(1,3-dioxolan-2-yl)benzoic acid (**3**)[13] (3.0 eq) was conjugated in the presence of HBTU (3.0 eq), HOBt•H_2O (3.0 eq), and DIEA (6.0 eq) in NMP. Finally, cleavage of the compound from the resin was performed using a cocktail (7:2:1 CH_2Cl_2/trifluoroethanol (TFE)/AcOH) at rt for 1.5 h. BPmoc-F$_3$ was obtained

after the deprotection with $NaIO_4$ and THF/H_2O (4:1 (vol/vol)). For Ald-F(F)F, deprotection was carried out in 1:3 mixture of 1 M HCl aq. and TFA at rt for 3 h. The crude product was purified by RP-HPLC (column: YMC ODS-A, gradient solvent: A: CH_3CN with 0.1% TFA, B: H_2O with 0.1% TFA) to give a target.

BPmoc-F_3[1]

White solid, 1H NMR (400 MHz, DMSO-*d6*): δ 8.30 (d, *J* = 8.0 Hz, 1H), 8.03 (d, *J* = 8.4 Hz, 1H), 8.00 (s, 2H), 7.71 (d, *J* = 8.0 Hz, 2H), 7.39 (d, *J* = 9.2 Hz, 1H), 7.12–7.28 (m, 17H), 4.84–4.94 (m, 2H), 4.52–4.59 (m, 1H), 4.41–4.48 (m, 1H), 4.15–4.23 (m, 1H), 2.72–3.09 (m, 5H) and 2.55–2.65 (m, 1H).

NPmoc-F(F)F[2]

White solid, 1H NMR (400 MHz, DMSO-*d6*): δ 8.26 (d, *J* = 8.4 Hz, 1H), 8.15 (d, *J* = 8.4 Hz, 2H), 7.63 (d, *J* = 8.8Hz, 1H), 7.43 (d, *J* = 8.8 Hz, 2H), 7.02–7.28 (m, 9H), 5.06 (s, 2H), 4.42 (m, 1H), 4.24 (m, 1H) and 2.64–3.08 (m, 4H).

Ald-F(F)F[3]

White solid. 1H NMR (600 MHz, DMSO-*d6*): δ 10.07 (s, 1H), 8.77 (d, *J* = 9.0 Hz, 1H), 8.39 (d, *J* = 9.0 Hz, 1H), 7.97 (d, *J* = 8.4 Hz, 2H), 7.93 (d, J = 8.4 Hz, 2H), 7.36 (dd, *J* = 8.4, 6.0 Hz, 2H), 7.24 (m, 4H), 7.18 (m, 1H), 7.07 (dd, *J* = 8.4 Hz, 2H), 4.75 (m, 1H), 4.48 (m, 1H), 3.08 (m, 2H), 2.93 (m, 2H).

Synthesis of the precursor for lipid-type gelators[4]

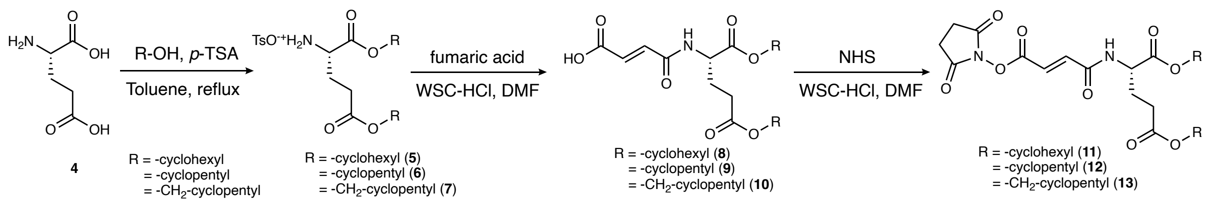

The precursor compounds were synthesized according to the previous report[4]. Briefly, ester compounds **5** to **7** were obtained from glutamic acid and corresponding alcohol in the presence of p-toluene sulfonic acid (*p*-TSA). A solution of water soluble carbodiimide (WSC)-HCl (1 eq) in N,N-dimethylformamide (DMF) was added dropwise to a stirring solution of fumaric acid (4 eq) and compound **5** to **7** (1 eq) in anhydrous DMF at room temperature. After completion of the reaction, the solvent was removed under reduced pressure at 50 °C. The residue was dissolved in ethyl acetate and the solution was washed 3 times with 5% critic acid solution. The organic layer was collected and dried over anhydrous Mg_2SO_4 and the solvent was evaporated to dryness. To remove excess amount of fumaric acid, the obtained oil was extracted with chloroform, and the filtrate was evaporated to yield crude **8** to **10**, which was used without further purification. To a solution of crude compound **8** to **10** in DMF was added N-hydroxysuccinimide (NHS) and WSC-HCl. The solution was stirred for 12 h at room temperature under N_2 atmosphere and the solvent was removed under reduced pressure at ambient temperature. The crude residue was dissolved in ethyl acetate and washed with brine. The residue was dried over anhydrous Mg_2SO_4, and the solvent was evaporated *in vacuo* and then the obtained oil was purified by column chromatography to give compounds **11** to **13**.

GalNAc-cycC$_6$[4]

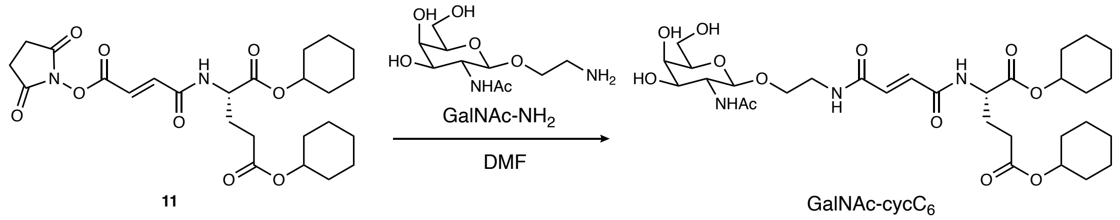

GalNAc-NH$_2$ (1 eq) was added to a stirring solution of **11** (1.05 eq) in DMF at 35 °C for 12 h.

The solvent was removed by evaporation at 50 °C, and the crude compound was washed with methanol several times and ion-exchanged water three times and dried for 12 h at 40 °C to give GalNAc-cycC$_6$ as a white solid. 1H-NMR (400 MHz, $CDCl_3/CD_3OD$): δ 6.87-6.93 (m, 2H), 4.74-4.82 (m, 2H), 4.56-4.58 (m, 1H), 4.41 (d, J = 8.4 Hz, 1H), 3.80-3.89 (m, 6H), 3.47-3.77 (m, 4H), 2.36-2.46 (m, 2H), 2.17-2.26, 1.98-2.09, 1.70-1.90 (m, 2H), 2.01 (s, 1H), 1.29-1.56 (m, 20H). *The reagent lot synthesized in the previous report[4] was used in this study.

Lys-cycC$_5$[5]

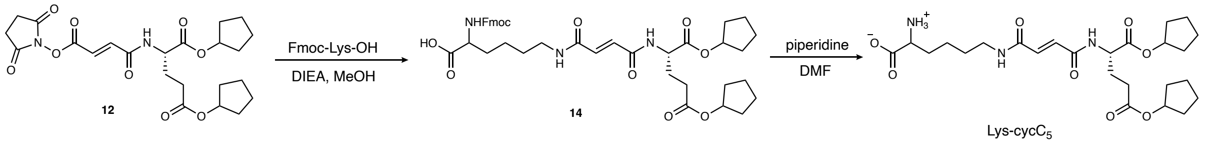

To a solution of **12** (1 eq) in dry MeOH was added Fmoc-Lys-OH (1.2 eq) and DIEA (2.7 eq) and the reaction mixture was stirred at room temperature for 20 min. After removal of the solvent, ethyl acetate was added to the residue and washed with 5% aqueous citric acid. The organic layer was dried over anhydrous $MgSO_4$ and evaporated to dryness. The residue was purified by column chromatography (silica gel, $CHCl_3$/MeOH = 20/1 + AcOH) to give **14** yield as a white solid. To a solution of **14** in DMF was added piperidine, and the reaction mixture was stirred at room temperature for 10 min. After removal of the solvent, the residue was washed with diethyl ether to give Lys-cycC$_5$ (88%) yield as a white solid. 1H NMR (400 MHz, CD_3OD): δ 6.92 (d, J = 14.8 Hz, 1H), 6.90 (d, J = 14.8 Hz, 1H), 5.19 (m, 1H), 5.15 (m, 1H), 4.45 (dd, J = 5.6 Hz, 1H), 3.53 (dd, J = 5.2 Hz, 1H), 3.31 (br, 2H), 2.39 (dd, J = 7.2 Hz, 2H), 2.14 (m, 1H), 1.94 (m, 1H), 1.45-1.98 (m, 22H). *The reagent lot synthesized in the previous report[5] was used in this study.

Phos-MecycC$_5$[6]

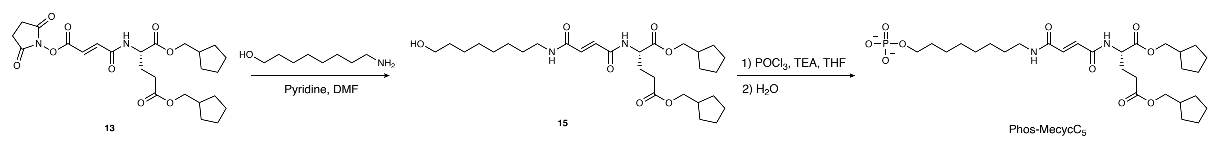

To a solution of **13** (1 eq) and 8-amino-1-octanol (1.2 eq) in dry DMF was added dry pyridine, and the reaction mixture was stirred at room temperature for 8 h under an argon atmosphere. After removal of solvent, the resulting oil was dissolved in chloroform and washed with 0.1 M aqueous HCl. The organic layer was dried over anhydrous $MgSO_4$, and the solvent was evaporated to dryness. The resulting solid was washed with diisopropyl ether and dried in vacuo to afford **15**. Under N_2 atmosphere, a solution of **15** (1 eq) and triethylamine (3.0 eq) in dry THF was added dropwise to a solution of distilled phosphoryl chloride (10 eq) in dry THF on an ice bath with stirring over 1.5 h. After removal of the precipitate, ion-exchanged water was added to the filtrate, and the resultant suspension was extracted with chloroform (80 mL × 3). The organic layer was dried over anhydrous $MgSO_4$, and the solvent was evaporated to dryness. The obtained gel-like residue was purified by RP-HPLC (column: YMC ODS-A, gradient solvent: A: CH_3CN with 0.1% TFA, B: H_2O with 0.1% TFA) and lyophilized to give Phos-MecycC$_5$ (28 mg, 37 %) as a white solid. 1H NMR (600 MHz, CD_3OD): δ 6.93 (d, J = 15.3 Hz, 1H), 6.88 (d, J = 15.3 Hz, 1H), 4.52 (q, J = 4.6 Hz, 1H), 4.07-4.00 (m, 2H), 3.97 (d, J = 7.2 Hz, 2H), 3.94 (q, J = 6.6 Hz, 2H), 3.26 (t, J = 7.2 Hz, 2H), 2.44 (t, J = 7.8 Hz), 2.24-2.17 (m, 3H), 2.02–1.98 (m, 1H), 1.76–1.72 (m, 4H), 1.66–1.55 (m, 12H), 1.42–1.23 (m, 12H).

DBS-COOH[7]

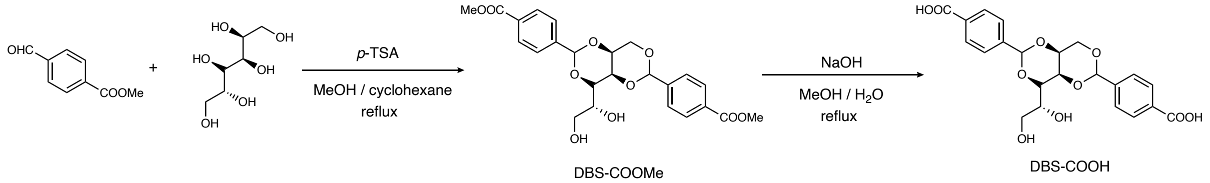

D-Sorbitol (490 mg) was weighed into a two-necked round-bottom flask with Dean–Stark apparatus. Cyclohexane (3.5 ml) and methanol (1 ml) were added, and the mixture was stirred

under N_2 at 50 °C for 20 min. 4-Methylcarboxylbenzaldehyde (750 mg) and p-toluene sulfonic acid hydrate (100 mg) was dissolved in methanol (3 ml) and stirred for 20 min at room temperature, before being added dropwise to the D-sorbitol mixture. The reaction temperature was increased to 70 °C and stirred for 1.5 h until most of the solvent was removed. The white paste formed was washed with methanol (10 ml × 3). The crude product was dried in vacuo. Mono- and tri-substituted derivatives were removed by washing with boiling water and boiling toluene, respectively to give DBS-COOMe (619 mg, 57%). DBS-COOMe (298 mg) was dissolved in methanol (6 ml), and NaOH(aq) (6 ml, 1 M) was added to the solution. The mixture was heated to reflux for 16 h. The mixture was neutralized with 1M HCl and was added with water (50 mL). The precipitate was centrifuged and washed with water (50 ml × 2). The product was dried *in vacuo* to give DBS-COOH (86.1 mg, 31%, white solid). 1H NMR (400 MHz, DMSO-*d*6): δ 13.03 (s, 2H), 7.97–7.95 (m, 4H), 7.60–7.56 (m, 4H), 5.75 (s, 2H), 4.91 (s, 1H), 4.47 (s, 1H), 4.23–4.20 (m, 3H), 4.00–3.95 (m, 1H), 3.91–3.83 (m, 1H), 3.80 (m, 1H), 3.61–3.57 (m, 1H), 3.48–3.44 (m, 1H). 1H-NMR was identical to that in the previous report[7].

TMR-Gua[8]

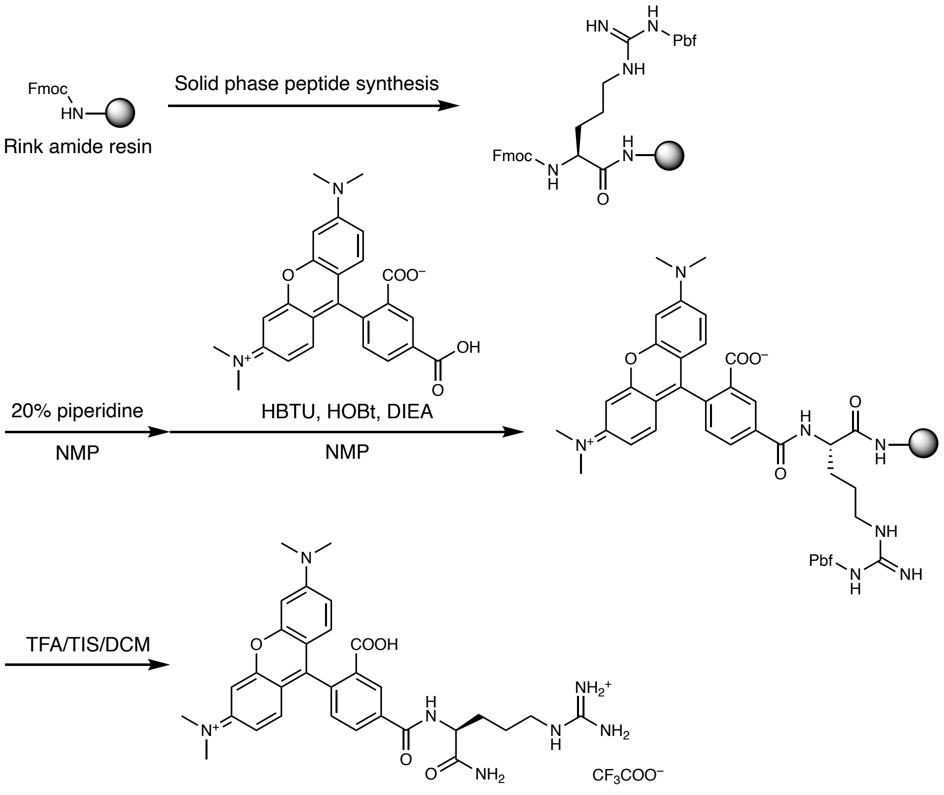

TMR-Gua

TMR-Gua was synthesized by solid-phase peptide synthesis using a commercially available rink amide resin (51.0 μmol). The condensation reaction was carried out in the presence of Fmoc-protected amino acids (3.0 eq), HBTU (3.0 eq), HOBt·H_2O (3.0 eq) and DIEA (6.0 eq) in NMP. Removal of Fmoc protecting group was performed using NMP solution containing 20% piperidine. After removal of the Fmoc group on the terminal amino group, the resulting free amino group was allowed to react with 5-carboxyl tetramethylrhodamine (3.0 eq) in the presence of HBTU (3.0 eq), HOBt·H_2O (3.0 eq), and DIEA (6.0 eq) in NMP. Finally, cleavage of the compound from the resin was performed using a cocktail (95:2.5:2.5 TFA/TIS/DCM) at rt for 1.5 h to give crude product. The crude product was purified by RP-HPLC (column: ODS-A, solvent gradient: A:B = 5:95 to 45:55 for 40 min, retention time: 32 min, A: CH_3CN with 0.1% TFA, B: H_2O with 0.1% TFA) to obtain TMR-Gua TFA salt as a red powder. 1H NMR (400 MHz, CD_3OD): δ 8.83 (d, J = 2.4 Hz, 1H), 8.32 (dd, J = 8.0, 2.4 Hz, 1H), 7.55 (d, J = 8.0 Hz, 1H), 7.14 (d, J = 9.6 Hz, 2H), 7.06 (dd, J = 9.6, 2.4 Hz, 2H), 7.00 (d, J = 2.4 Hz, 2H), 4.73–

4.64 (m, 1H), 2.13–1.83 (m, 2H), 1.82–1.62 (m, 2H). *The reagent lot synthesized in the previous report[8] was used in this study.

Alx546-cycC$_6$[9]

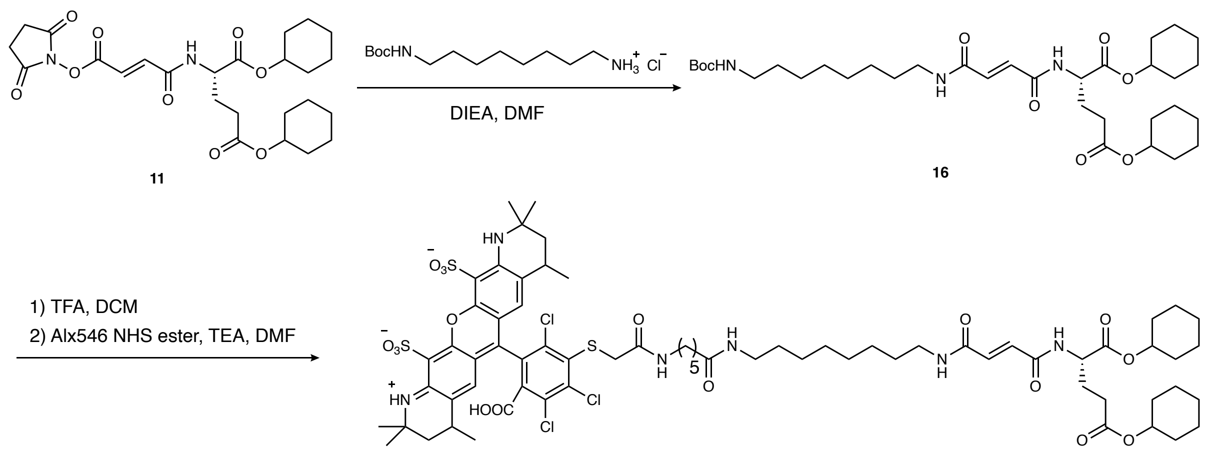

To a solution of DMF, **11** (1.0 eq) and 8-((tert-butoxycarbonyl)amino)octan-1-aminium chloride (1.4 eq) was added DIEA (3.9 eq). The mixture was stirred overnight at room temperature. $CHCl_3$ was added to the reaction mixture and the solution was washed with 5% aqueous citric acid, saturated $NaHCO_3$, water, and brine. The organic layer was dried over Na_2SO_4, and the filtrate was evaporated to remove the solvent. The crude product was purified by column chromatography (silica, $CHCl_3$/AcOEt = 6:1) to give **16** as a white solid (76%). A mixture of CH_2Cl_2, **16**, and TFA was stirred at room temperature for 2.5 h. The volatile was removed under reduced pressure and by azeotropy with toluene. To the residue was added DMF solution of Alexa546 NHS ester (1.0 eq) and TEA (10 eq) under N_2 atmosphere. The solution was stirred at room temperature for 4 h. The solvent was removed and the resultant residue was purified by RP-HPLC (solvent gradient: CH_3CN/H_2O (0.1% TFA) from 20:80 to 80:20 for 40 min) to give Alexa546-cycC$_6$ as a pink solid (25%). 1H-NMR (CD_3OD, 400 MHz): δ 7.02–7.08 (m, 2H), 6.91–6.96 (d, J = 14.8 Hz, 1H), 6.87–6.91 (d, J = 14.8 Hz, 1H), 4.52–4.67 (m, 1H), 3.61–3.63 (d, J = 11.2 Hz, 2H), 2.90–3.01 (m, 2H), 2.40–2.44 (t, J = 7.6 Hz, 2H), 2.05–2.20 (m, 2H), 1.85–1.96 (m, 2H), 1.79–1.84 (br, 4H), 1.68–1.79 (br, 4H), 1.22–1.60 (m, 48H).

*The reagent lot synthesized in the previous report[9] was used in this study.

Agarose-Alx488[10]

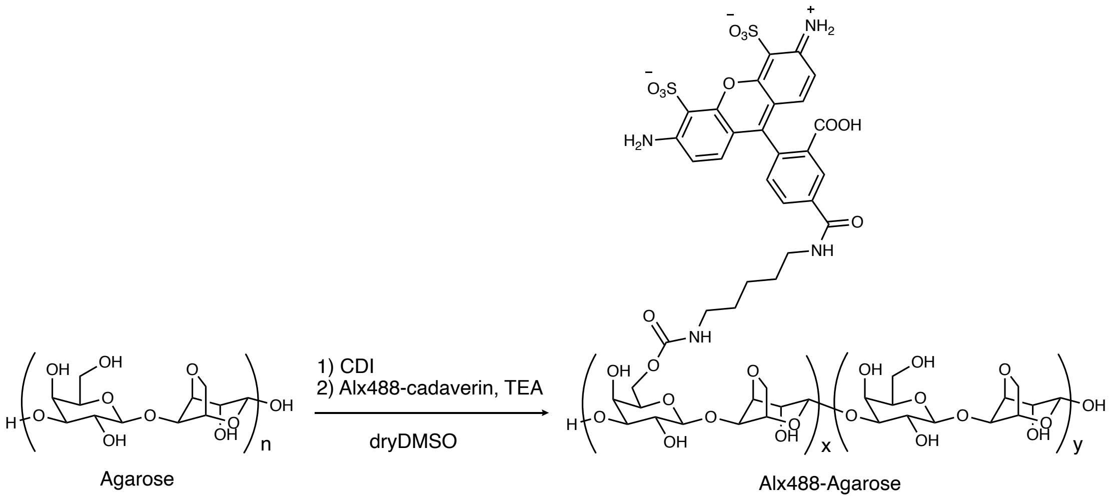

To a dry DMSO solution of agarose (1.0 eq as monomer unit) was added a DMSO solution of carbonyldiimidazole (0.1 M, 0.2 eq, CDI). The reaction mixture was allowed to stir at rt for 2 h. To this mixture, a DMSO solution of Alx488-cadaverin (0.0025 eq) and TEA (0.06 eq) was added. The resultant mixture was stirred at rt for 24 h. After then, the mixture was dialyzed in H_2O (1 L×6, MWCO 3500). The mixture was lyophilized to obtain Alx488-Agarose as a pale yellow solid. The modification ratio (0.023 mol%) was determined by UV/Vis absorption spectroscopy. *The reagent lot synthesized in the previous report[10] was used in this study.

The quantitative analyses of heterogeneity of the agarose network. The 16-bit images of the Alx488 channel were standardized with saturated pixel at 1%, and then the standard deviations were calculated by Histogram program in Fiji.

Network pattern classification. According to our quantitative image analyses, the network patterns can be classified based mainly on Pearson's correlation coefficient values, the standard deviation value and the island/void sizes of the agarose network. We can set the criteria of PCC value at 0.2 to differentiate the network patterns: If the value of Pearson's correlation

coefficient is lower than 0.2, the composite hydrogels were classified into orthogonal or interactive type II; otherwise, interactive type I or III. The network patterns are further divided by use of the standard deviation value and the island/void sizes of the agarose network. Compared to the single-component agarose gel, the network can be classified into the interactive type I or II if those values are significantly smaller; otherwise, orthogonal or interactive type III (in the interactive type I, the agarose network obviously changes into the fibrous structure). It has not been confirmed whether this classification can be applicable as a universal guide because of some exceptional composite hydrogels and the small sample size. For example, the BPmoc-F_3/agarose hydrogel prepared by the pH decreasing protocol shows an intermediate network that exhibits characteristics of both orthogonal and interactive III networks. Therefore, the network classification should be carefully conducted by combination of both quantitative analysis of the network structure and in-depth observation of the network formation process, as described in the main text.

Rheological analyses. The hydrogels were prepared in the PDMS molds. The resultant disk-shaped hydrogels (diameter: *ca.* 10 mm; height: *ca.* 3 mm) were carefully taken out from the PDMS mold and put onto the stage of a rheometer (MCR-502, Anton Paar) with a parallel plate geometry. Linear dynamic viscoelasticity was measured in shear mode with a strain amplitude of 1.0%, and nonlinear dynamic viscoelasticity using strain amplitude as a variable was examined in shear mode with an angular frequency of 1.0 or 0.2 rad/s (for Phos-MecycC$_5$ gel).

The quantitative analyses of time-course change of network formation. The number of the nanofibers and the agarose domains in the images was counted according to a previous report.[15] The background was initially subtracted using the background subtraction tool with a rolling ball radius of 10 pixels. The image stack was then thresholded using the default thresholding tool and method, converting the stack to binary images. For the supramolecular network, the

Analyze Particles tool was used to count particles with a size of 0.1 μm^2 or greater and a circularity of 0.00 to 0.50, yielding particles in each time frame with an elongated ellipsoidal shape and a length of ~0.1 μm or greater. For the agarose network, the binary images were processed with the watershed, and Analyze Particles tool was used to count particles with a size of 0.5 μm^2 or greater and a circularity of 0.00 to 1.00, yielding particles in each time frame with a length of ~0.5 μm or greater.

CD spectroscopy. The hot solution (200 μL) of NPmoc-F(F)F (0.4 wt%, 7.9 mM) and agarose (0.5 wt%) was poured onto a piece of an assembled quartz cell (optical length: 0.5 mm) and incubated at rt for 1 h or 24 h in the presence of water to avoid dryness. The CD spectra were measured by using a JASCO J-1100WI spectrometer.

Supplementary figures

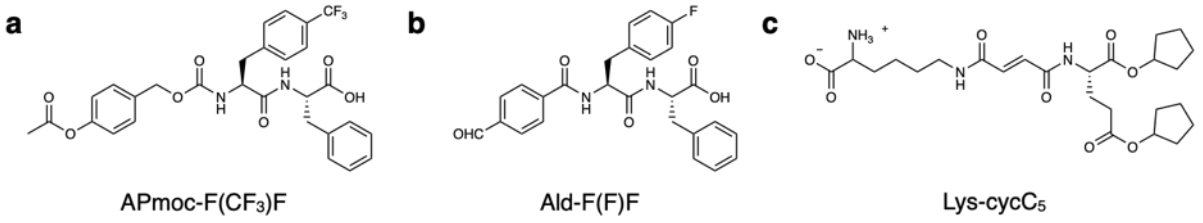

Supplementary Figure 1. Chemical structures of other gelators. (**a**) APmoc-F(CF_3)F, (**b**) Ald-F(F)F, and (**c**) Lys-cycC_5.

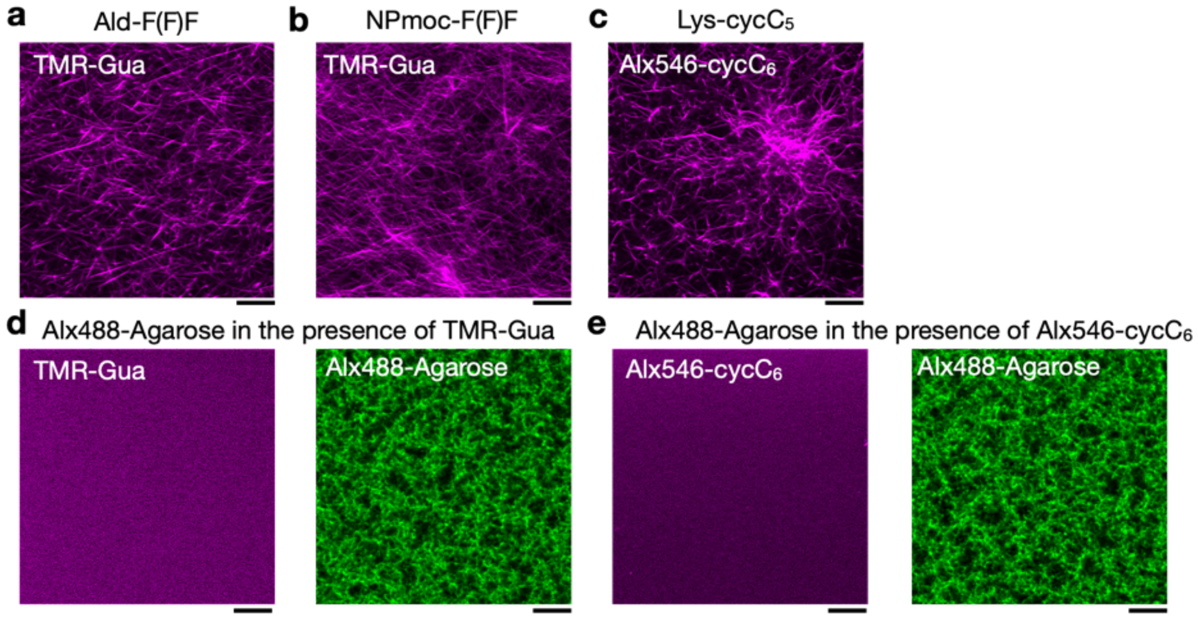

Supplementary Figure 2. CLSM images of single-component hydrogels and staining selectivity of the fluorescent probes. (**a** to **c**) CLSM images of (**a**) 0.4 wt% Ald-F(F)F, (**b**) 0.4 wt% NPmoc-F(F)F, and (**c**) 0.3 wt% Lys-cycC$_5$. (**d**, **e**) CLSM images of 0.5 wt% Alx488-Agarose in the presence of (**d**) TMR-Gua or (**e**) Alx546-cycC$_6$. No sructures were observed in the TMR channel or the Alx546 channel, suggesting the fluorescent probes can selectively stain the supramolecular fibers. Conditions: [Ald-F(F)F] = 0.4 wt% (8.6 mM), [NPmoc-F(F)F] = 0.4 wt% (7.9 mM), [Lys-cycC$_5$] = 0.3 wt% (5.9 mM), [Alx488-Agarose] = 0.5 wt%, [TMR-Gua] = 14 µM, [Alx546-cycC$_6$] = 4 µM, solvent: 100 mM MES pH 7.0, scale bar: 5 µm, rt.

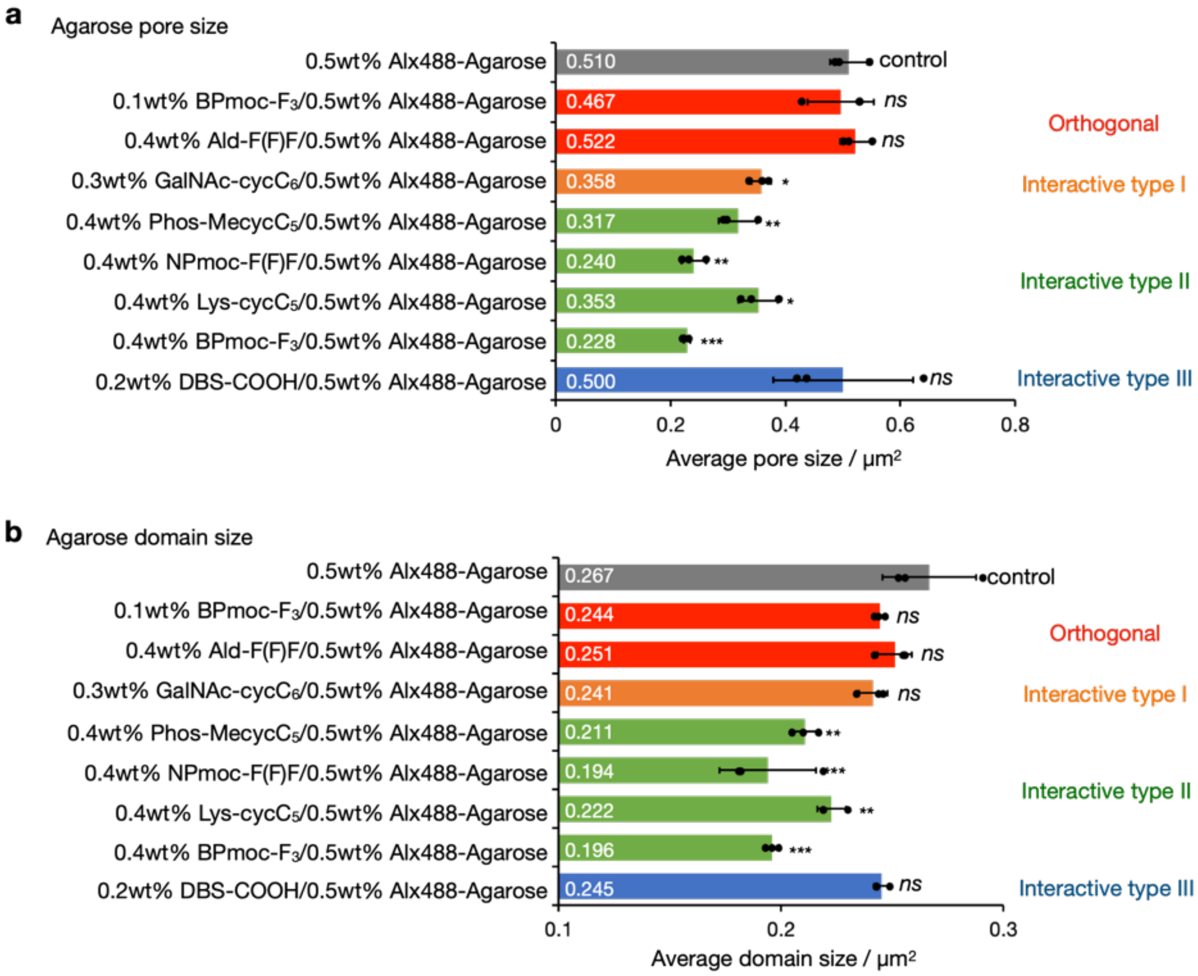

Supplementary Figure 3. Quantitative analyses of the pore size and the island domain size of the agarose network. (**a**) The average pore size, and (**b**) the island domain size of agarose network. The data present the mean ± s.d. ($n = 3$). *Post hoc* multiple comparisons were conducted using a one-way ANOVA followed by Dunnett's test. ***: $P < 0.001$, **: $P < 0.01$, *: $P < 0.05$, *ns*: no significant difference. Conditions: [BPmoc-F_3] = 0.1 wt% (1.6 mM), 0.4 wt% (6.4 mM), [NPmoc-F(F)F] = 0.4 wt% (7.0 mM), [Ald-F(F)F] = 0.4 wt% (8.6 mM), [Phos-MecycC$_5$] = 0.4 wt% (6.5 mM), [Lys-cycC$_5$] = 0.3 wt% (5.9 mM), [GalNAc-cycC$_6$] = 0.3 wt% (4.6 mM), [DBS-COOH] = 0.2 wt% (4.5 mM), [TMR-Gua] = 14 μM, [Alx546-cycC$_6$] = 4 μM, [Alx488-Agarose] = 0.5 wt%, [glucono-δ-lactone] = 44.9 mM, solvent: 100 mM MES pH 7.0 (except for DBS-COOH) or water (DBS-COOH).

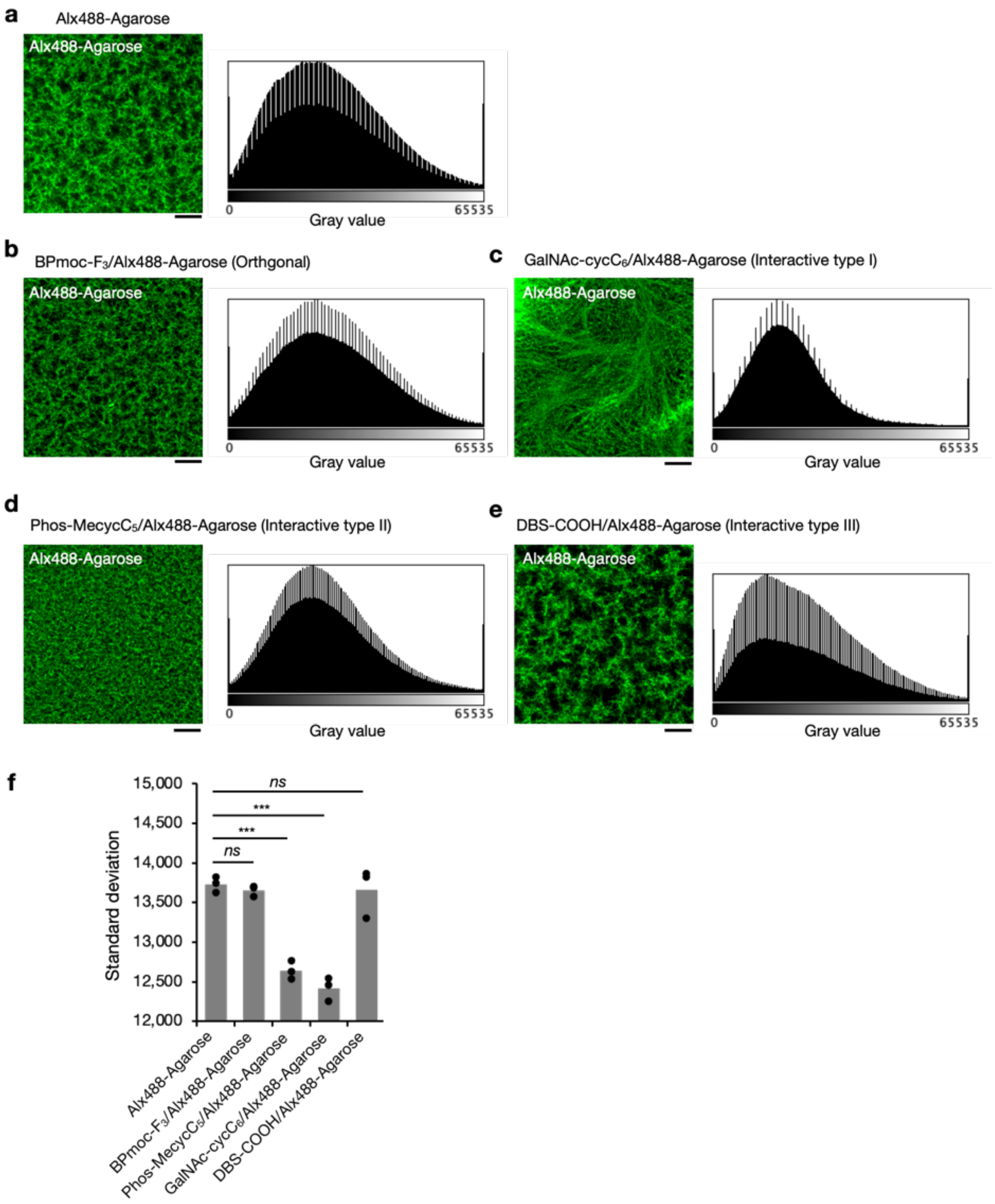

Supplementary Figure 4. Histogram analyses of heterogeneity of the agarose network. (**a** to **e**) (left) CLSM images and (right) the fluorescence intensity distribution of the Alx488 channel in the single component agarose gel or the composite hydrogels. (**a**) Single-component Alx488-Agarose, (**b**) BPmoc-F_3/Alx488-Agarose, (**c**) GalNAc-cycC_6/Alx488-Agarose, (**d**) Phos-MecycC_5/Alx488-Agarose, (**e**) DBS-COOH/Alx488-Agarose. (**f**) Standard deviations of the fluorescence intensity distribution in the Alx488 channel (n = 3). *Post hoc* multiple comparisons were conducted using a one-way ANOVA followed by Dunnett's test. ***: $P <$

0.001, *ns*: no significant difference. Conditions: [BPmoc-F_3] = 0.1 wt% (1.6 mM), [Phos-MecycC_5] = 0.4 wt% (6.5 mM), [GalNAc-cycC_6] = 0.3 wt% (4.6 mM), [Alx488-Agarose] = 0.5 wt%, [TMR-Gua] = 14 μM, [Alx546-cycC_6] = 4 μM, [DBS-COOH] = 0.2 wt% (4.5 mM), [glucono-δ-lactone] = 44.9 mM (for DBS-COOH), solvent: 100 mM MES pH 7.0 (for **a** to **d**) or water (for DBS-COOH), rt.

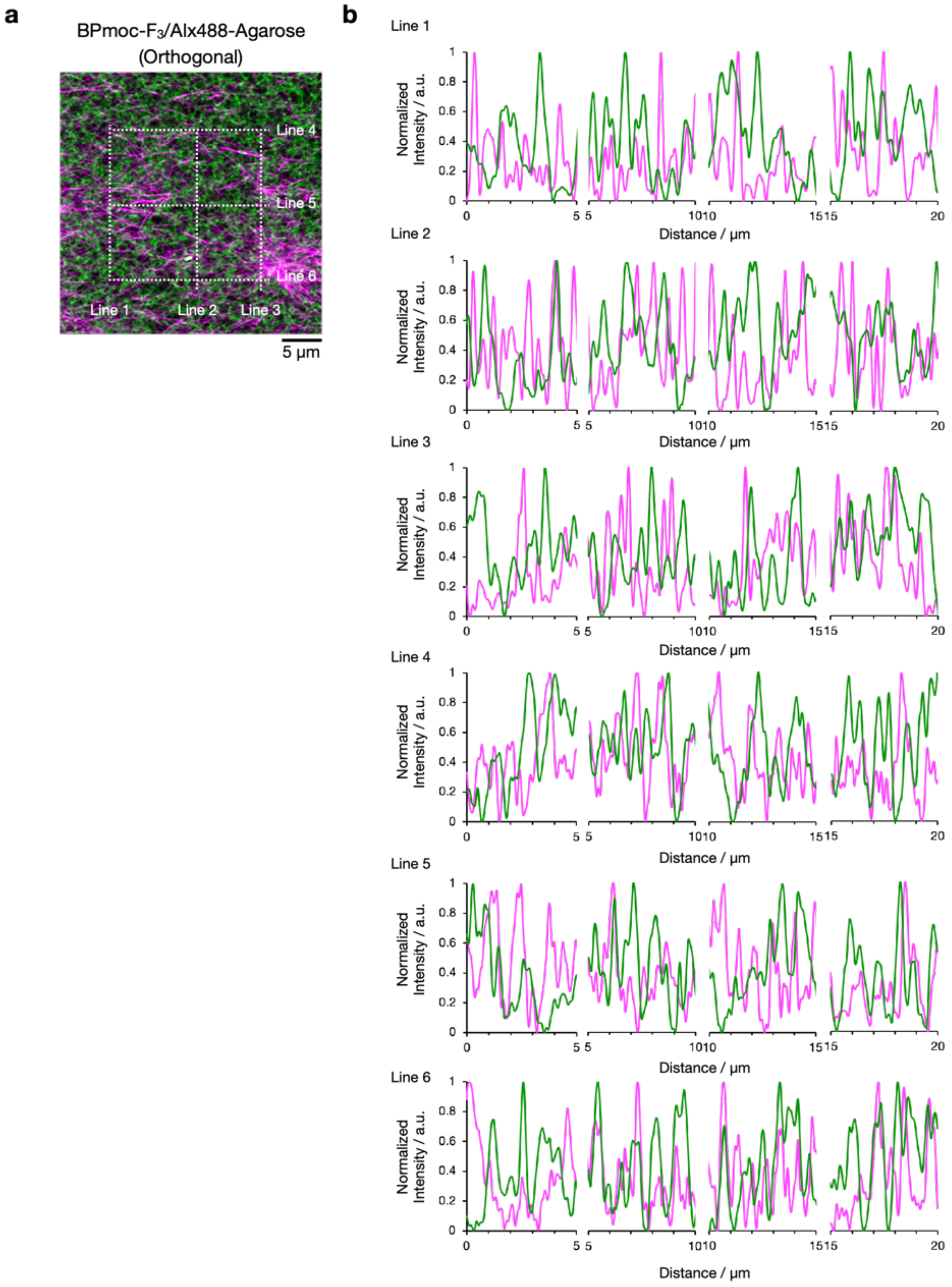

Supplementary Figure 5. Line plot analyses of orthogonal network. (**a**) CLSM image of the BPmoc-F_3/Alx488-Agarose showing the orthogonal network (same as Figure 2b). Magenta: TMR-Gua, Green: Alx488-Agarose. (**b**) Line plot analyses along white lines shown in Supplementary Fig. 5a. To compare the peak tops of the supramolecular and agarose

networks, the peak intensity was normalized at 5 μm intervals so that the maximum and minimum intensities were set to 1 and 0, respectively. a.u.: arbitrary units.

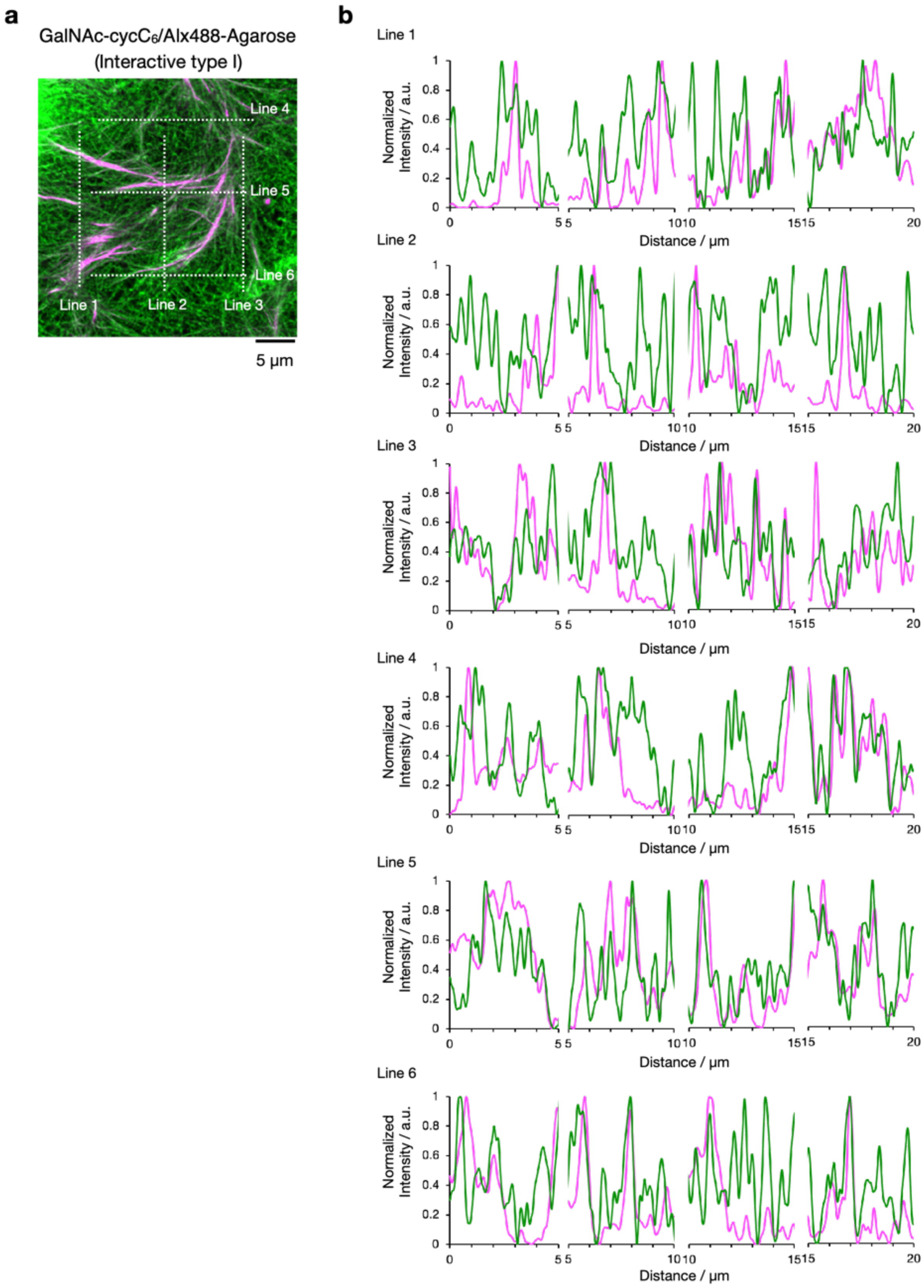

Supplementary Figure 6. Line plot analyses of interactive type I network. (**a**) CLSM image of the GalNAc-cycC$_6$/Alx488-Agarose showing the interactive type I network (same as Figure 2d). Magenta: TMR-Gua, Green: Alx488-Agarose. (**b**) Line plot analyses along white lines

shown in Supplementary Fig. 6a. To compare the peak tops of the supramolecular and agarose networks, the peak intensity was normalized at 5 μm intervals so that the maximum and minimum intensities were set to 1 and 0, respectively. a.u.: arbitrary units.

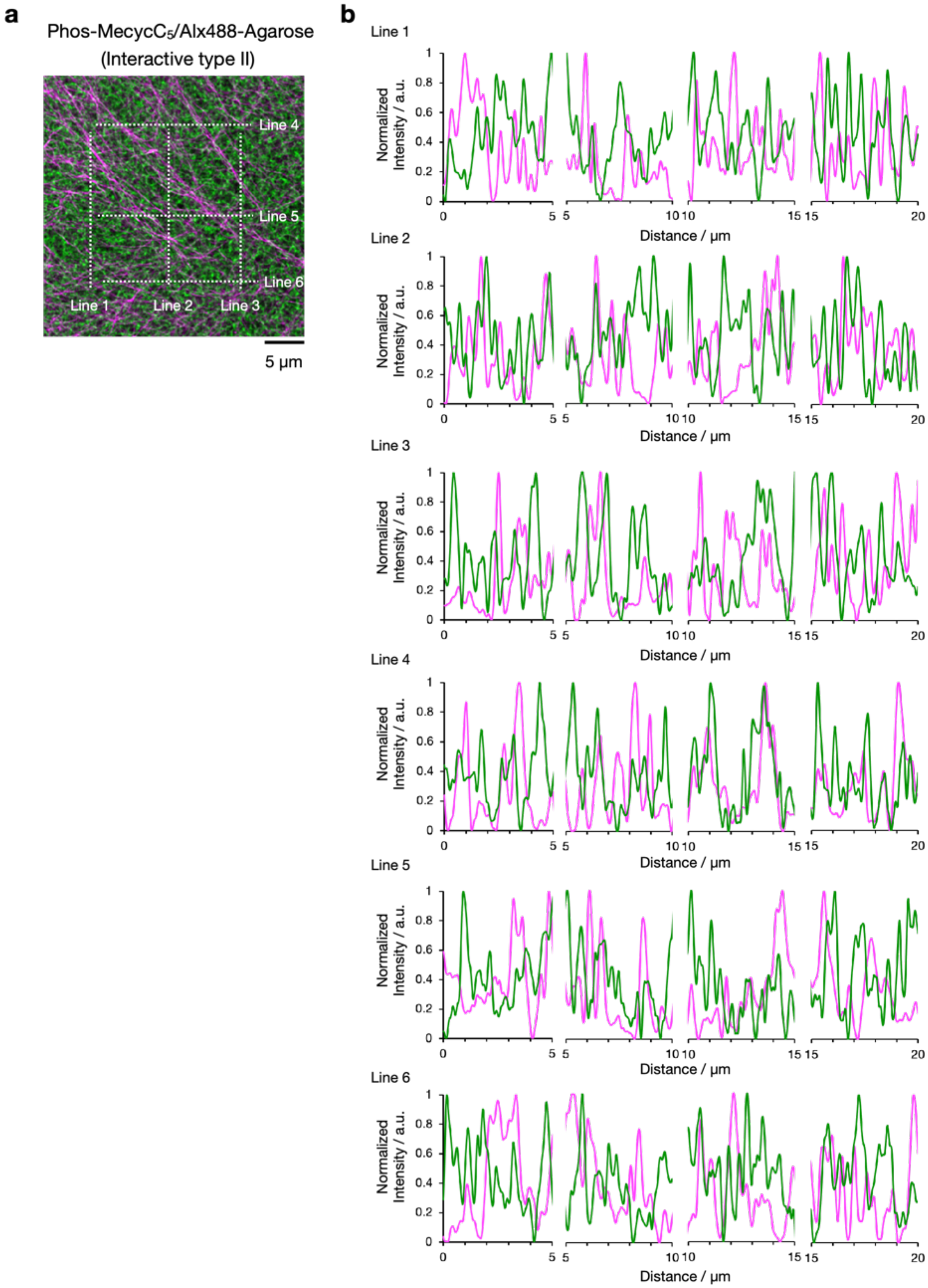

Supplementary Figure 7. Line plot analyses of interactive type II network. (**a**) CLSM image of the Phos-MecycC$_5$/Alx488-Agarose showing interactive type II network (same as Figure 2f). Magenta: TMR-Gua, Green: Alx488-Agarose. (**b**) Line plot analyses along white

lines shown in Supplementary Fig. 7a. To compare the peak tops of the supramolecular and agarose networks, the peak intensity was normalized at 5 μm intervals so that the maximum and minimum intensities were set to 1 and 0, respectively. a.u.: arbitrary units.

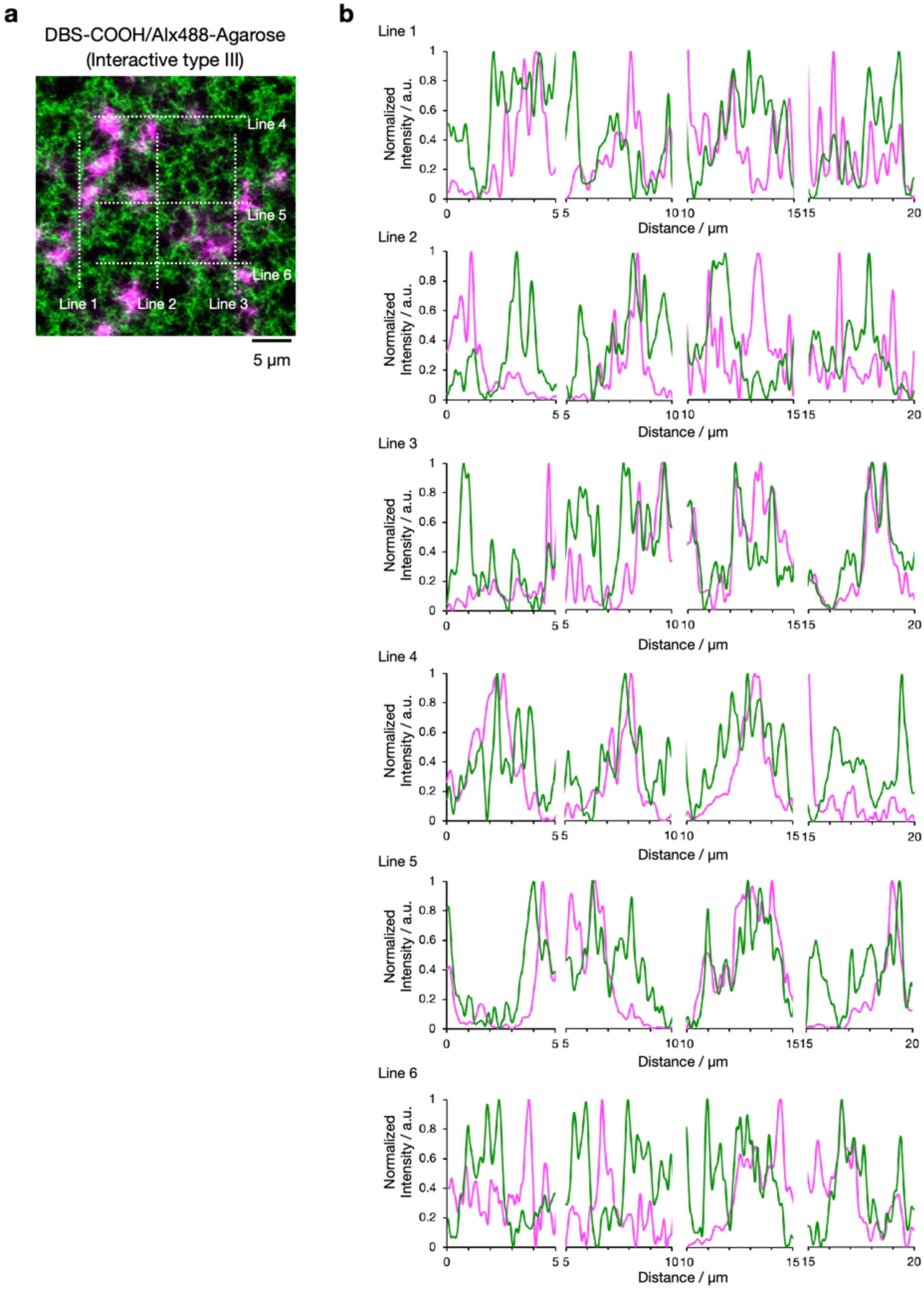

Supplementary Figure 8. Line plot analyses of interactive type III network. (**a**) CLSM image of the DBS-COOH/Alx488-Agarose showing interactive type III network (same as Figure 2h). Magenta: TMR-Gua, Green: Alx488-Agarose. (**b**) Line plot analyses along white

lines shown in Supplementary Fig. 8a. To compare the peak tops of the supramolecular and agarose networks, the peak intensity was normalized at 5 μm intervals so that the maximum and minimum intensities were set to 1 and 0, respectively. a.u.: arbitrary units.

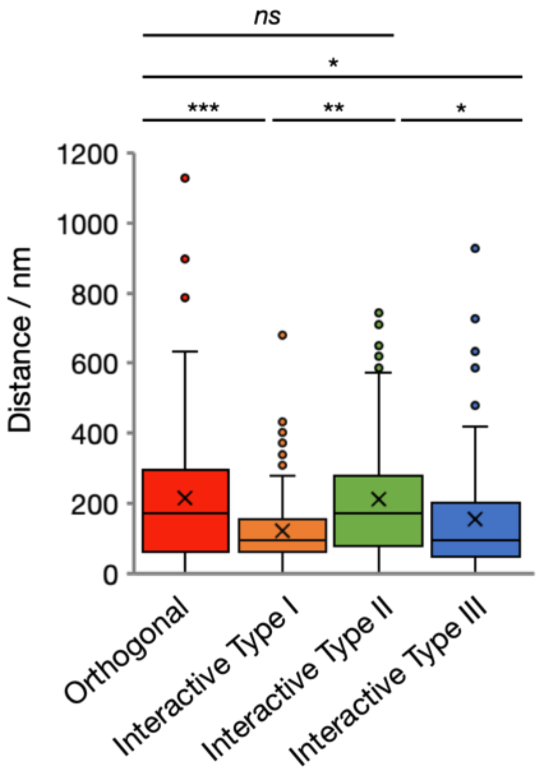

Supplementary Figure 9. Quantitative analysis of nearest peak distances between the supramolecular and agarose networks. Boxplot of the nearest peak distances. The box begins with the first quartile and ends with the third quartile. A line that divides the box: median, cross mark: average, upper whisker: maximum, lower whisker: minimum. dot: outlier. *Post hoc* multiple comparisons were conducted using a one-way ANOVA followed by Tukey's test. ***: $P < 0.001$, **: $P < 0.01$, *: $P < 0.05$, *ns*: no significant difference.

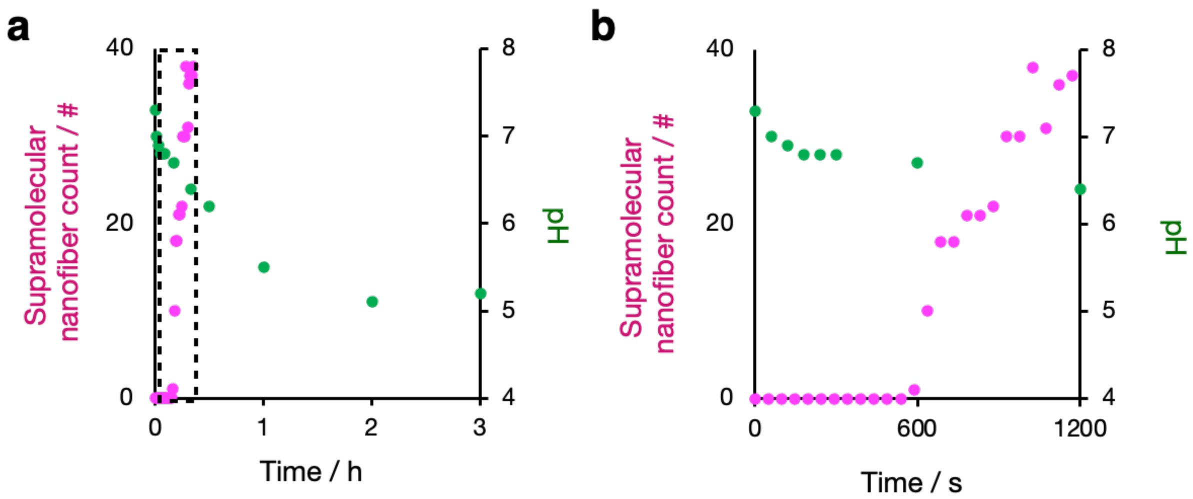

Supplementary Figure 10. A pH change during glucono-δ-lactone hydrolysis. (**a**) Time plot of DBS-COOH formation (the same as Figure 4h) and pH in the composite hydrogel. Magenta: DBS-COOH formation, green: pH. (**b**) Magnified view for 0 to 1200 s.

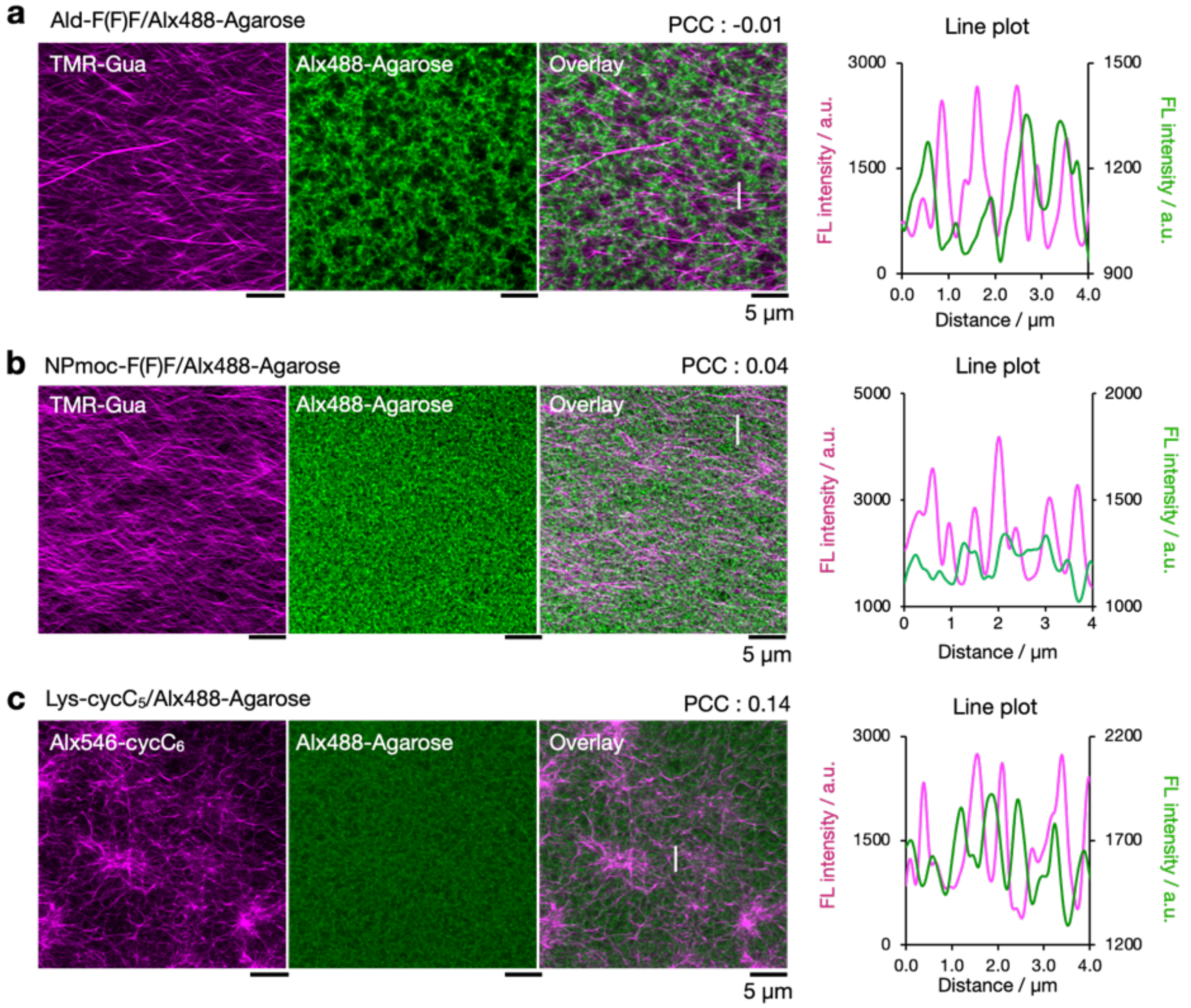

Supplementary Figure 11. CLSM imaging of composite hydrogels composed of other supramolecular gelators and Alx488-Agarose. (**a**) Ald-F(F)F/Alx488-Agarose, (**b**) NPmoc-F(F)F/Alx488-Agarose, and (**c**) Lys-cycC$_5$/Alx488-Agarose. Magenta: supramolecular network, green: agarose. The right figures are line plot analyses along the white line shown in the merged images. PCC: Perason's correlation coefficient, FL intensity: fluorescence intensity, a.u.: arbitrary units. Conditions: [Ald-F(F)F] = 0.4 wt% (8.6 mM), [NPmoc-F(F)F] = 0.4 wt% (7.9 mM), [Lys-cycC$_5$] = 0.3 wt% (5.9 mM), [TMR-Gua] = 14 μM, [Alx546-cycC$_6$] = 4 μM, [Alx488-Agarose] = 0.5 wt%, solvent: 100 mM MES pH 7.0, scale bar: 5 μm, rt.

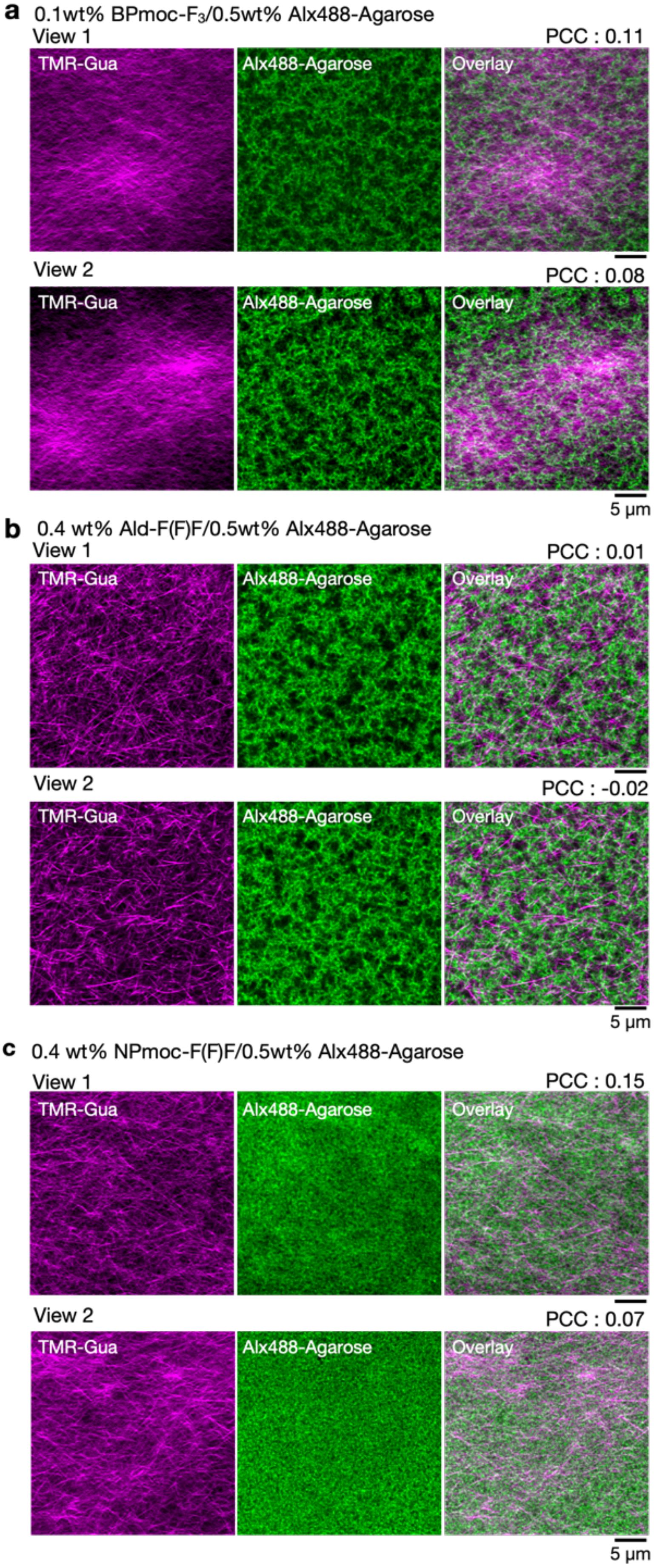
a 0.1wt% BPmoc-F3/0.5wt% Alx488-Agarose
View 1
PCC : 0.11
TMR-Gua
Alx488-Agarose
Overlay
View 2
PCC : 0.08
TMR-Gua
Alx488-Agarose
Overlay
5 μm
b 0.4 wt% Ald-F(F)F/0.5wt% Alx488-Agarose
View 1
PCC : 0.01
TMR-Gua
Alx488-Agarose
Overlay
View 2
PCC : -0.02
TMR-Gua
Alx488-Agarose
Overlay
5 μm
c 0.4 wt% NPmoc-F(F)F/0.5wt% Alx488-Agarose
View 1
PCC : 0.15
TMR-Gua
Alx488-Agarose
Overlay
View 2
PCC : 0.07
TMR-Gua
Alx488-Agarose
Overlay
5 μm

Supplementary Figure 12. Additional views of CLSM images of the peptide/agarose composite hydrogels. (**a**) BPmoc-F_3/Alx488-Agarose, and (**b**) Ald-F(F)F/Alx488-Agarose, and (**c**) NPmoc-F(F)F/Alx488-Agarose. Magenta: TMR-Gua, green: Alx488-Agarose. PCC: Pearson's correlation coefficient, FL intensity: fluorescence intensity. Conditions: [BPmoc-F_3] = 0.1 wt% (1.6 mM) [Ald-F(F)F] = 0.4 wt% (8.6 mM), [NPmoc-F(F)F] = 0.4 wt% (7.9 mM), [TMR-Gua] = 14 μM, [Alx488-Agarose] = 0.5 wt%, solvent: 100 mM MES pH 7.0, scale bar: 5 μm, rt.

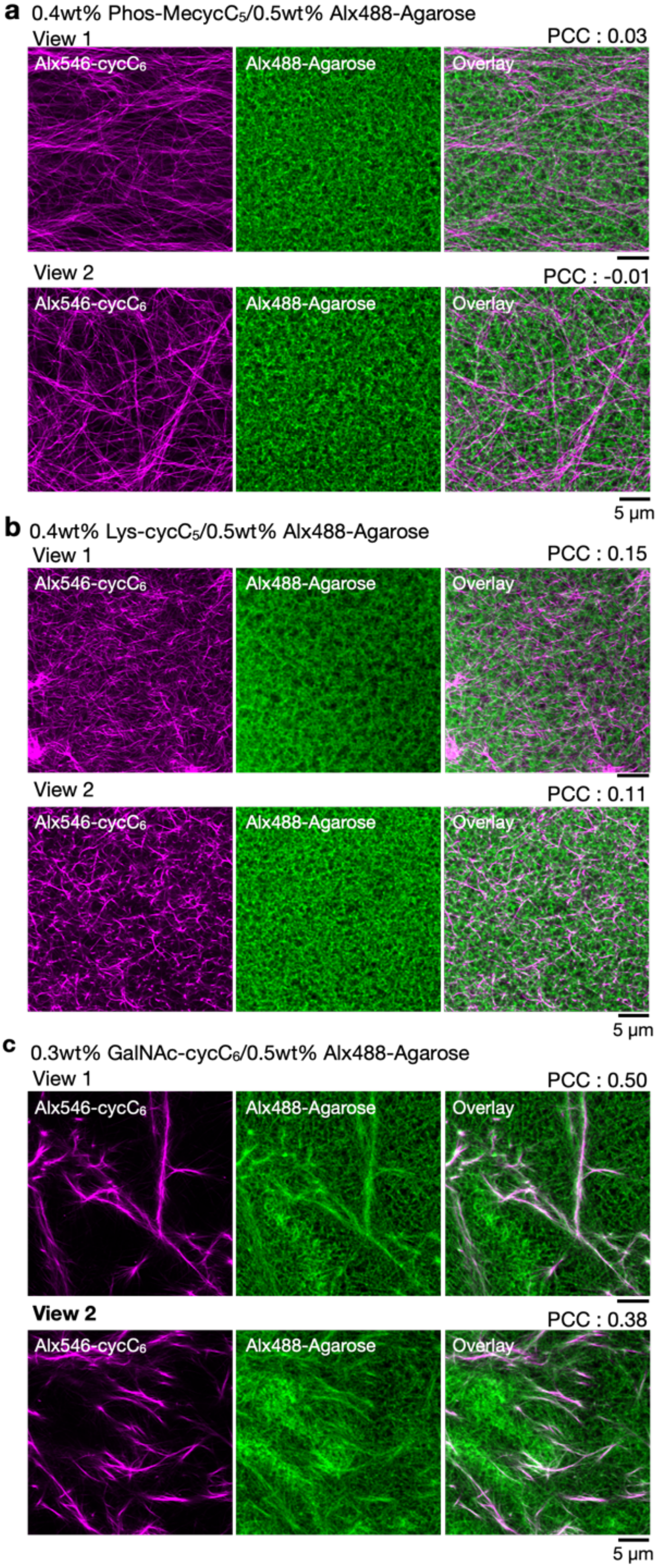
a
0.4wt% Phos-MecycC5/0.5wt% Alx488-Agarose
View 1
PCC : 0.03
Alx546-cycC6
Alx488-Agarose
Overlay
View 2
PCC : -0.01
5 μm
b
0.4wt% Lys-cycC5/0.5wt% Alx488-Agarose
View 1
PCC : 0.15
View 2
PCC : 0.11
5 μm
c
0.3wt% GalNAc-cycC6/0.5wt% Alx488-Agarose
View 1
PCC : 0.50
View 2
PCC : 0.38
5 μm

Supplementary Figure 13. Additional views of CLSM images of the lipid-type gelators/agarose composite hydrogels. (**a**) Phos-MecycC_5/Alx488-Agarose, and (**b**) Lys-cycC_5/Alx488-Agarose, and (**c**) GalNAc-cycC_6/Alx488-Agarose. Magenta: Alx546-cycC_6, green: Alx488-Agarose. PCC: Pearson's correlation coefficient, FL intensity: fluorescence intensity. Conditions: [Phos-MecycC_5] = 0.4 wt% (6.5 mM), [GalNAc-cycC_6] = 0.3 wt% (4.6 mM), [Lys-cycC_5] = 0.4 wt% (5.9 mM), [Alx546-cycC_6] = 4 μM, [Alx488-Agarose] = 0.5 wt%, solvent: 100 mM MES pH 7.0, scale bar: 5 μm, rt.

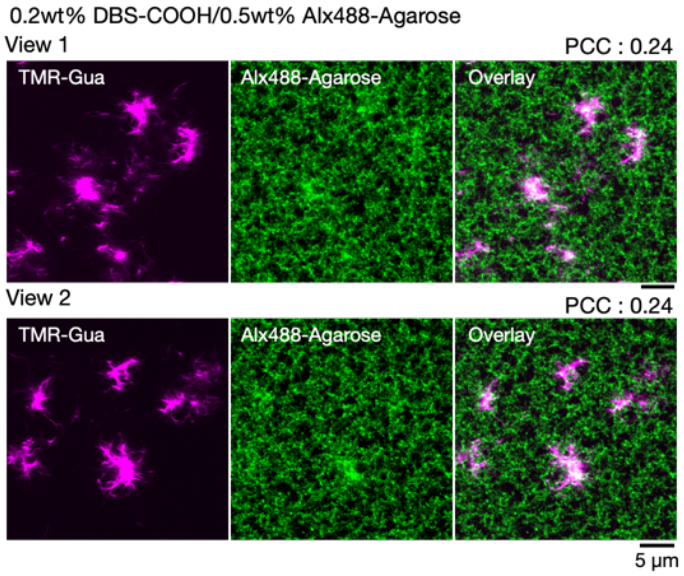

Supplementary Figure 14. Additional views of CLSM images of the sugar-type gelator/agarose composite hydrogel. DBS-COOH/Alx488-Agarose. Magenta: TMR-Gua, green: Alx488-Agarose. PCC: Perason's correlation coefficient, FL intensity: fluorescence intensity. Conditions: [DBS-COOH] = 0.2 wt% (4.5 mM), [Alx488-Agarose] = 0.5 wt%, [Glucono-δ-lactone] = 4.49 mM, solvent: water, scale bar: 5 μm, rt.

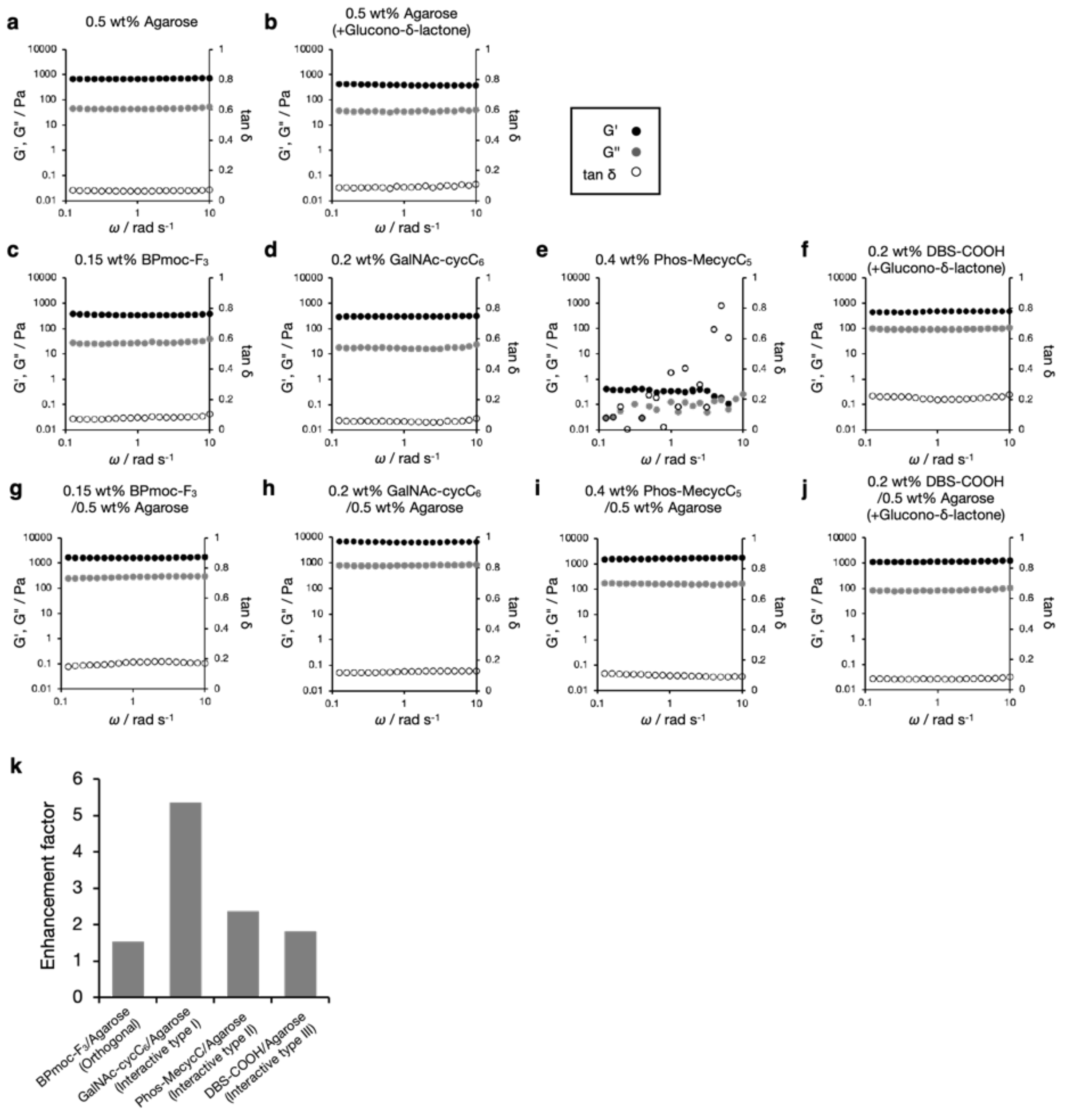

Supplementary Figure 15. Linear dynamic viscoelastic properties. (**a**) Agarose, (**b**) Agarose prepared by a pH decrease protocol, (**c**) BPmoc-F_3, (**d**) GalNAc-cycC_6, (**e**) Phos-MecycC_5, (**f**) DBS-COOH, (**g**) BPmoc-F_3/Agarose, (**h**) GalNAc-cycC_6/Agarose, (**i**) Phos-MecycC_5/Agarose, (**j**) DBS-COOH/Agarose. (**k**) Enhancement factor of each network pattern. The enhancement factor is defined as the ratio of the G' of the composite hydrogel over the sum of that of each single-component hydrogel. The tan δ values of the composite hydrogels were slightly higher than that of the single-component agarose hydrogel probably due to liquid-like property of supramolecular networks. Strain amplitude: 1.0%. G': storage shear modulus, G'': loss shear modulus. Conditions: [BPmoc-F_3] = 0.1 wt% (2.4 mM), [Phos-MecycC_5] = 0.4 wt% (6.5 mM), [GalNAc-cycC_6] = 0.2 wt% (3.1 mM), [Agarose] = 0.5 wt%, [DBS-COOH] = 0.2 wt% (4.5 mM), [glucono-δ-lactone] = 44.9 mM (for DBS-COOH), solvent: 100 mM MES

pH 7.0 (except for DBS-COOH) or water (for DBS-COOH), rt.

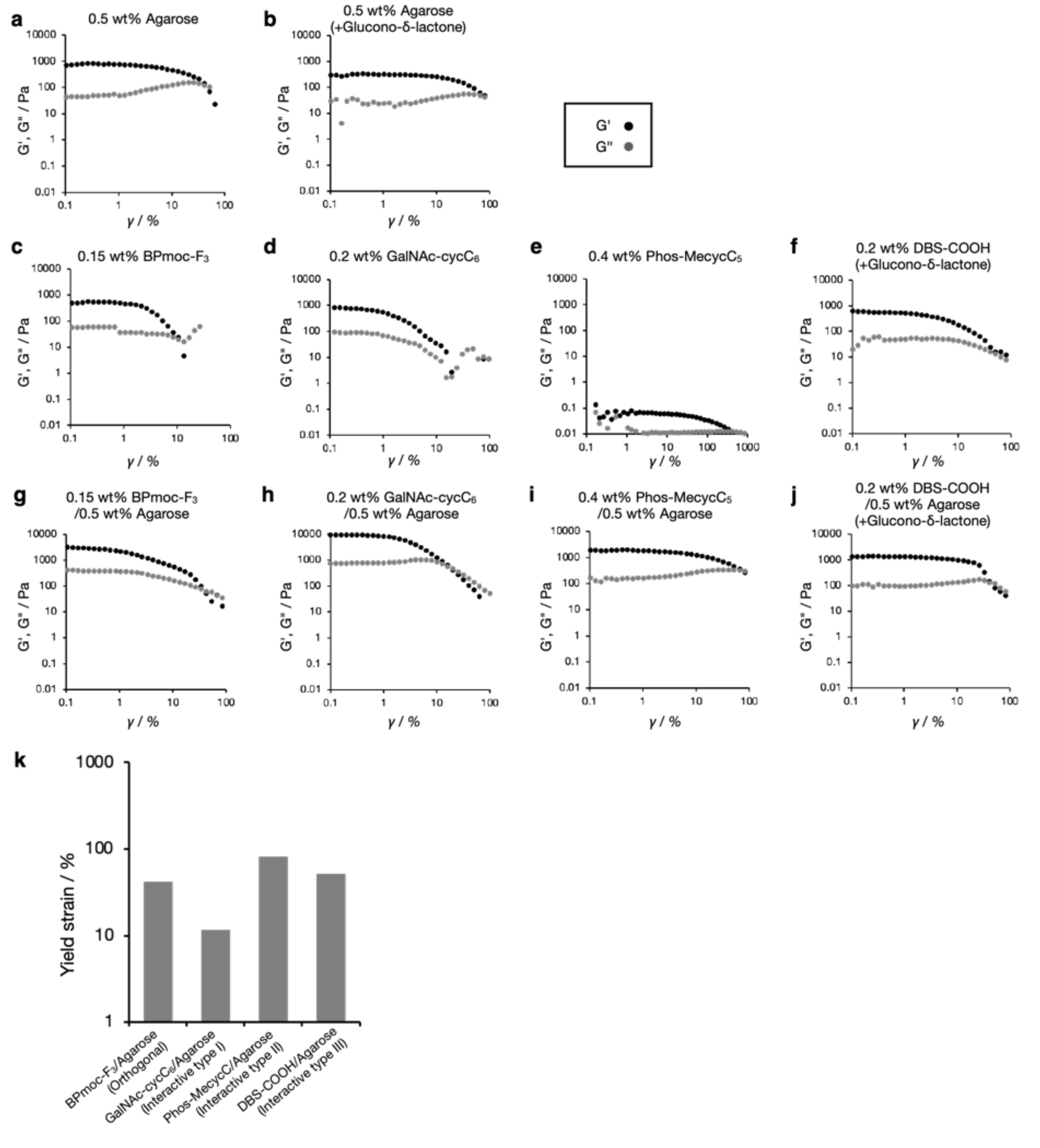

Supplementary Figure 16. Nonlinear viscoelastic properties investigated using large amplitude oscillatory shear. (**a**) Agarose, (**b**) Agarose prepared by a pH decrease protocol, (**c**) BPmoc-F_3, (**d**) GalNAc-cycC_6, (**e**) Phos-MecycC_5, (**f**) DBS-COOH, (**g**) BPmoc-F_3/Agarose, (**h**) GalNAc-cycC_6/Agarose, (**i**) Phos-MecycC_5/Agarose, (**j**) DBS-COOH/Agarose. (**k**) Yield strain of each network pattern. The yield strain is defined as the G'/G'' cross-over point. Angular frequency is 1.0 rad/s (except for **e**) or 0.2 rad/s (**e**). G': storage shear modulus, G'': loss shear modulus. Conditions: [BPmoc-F_3] = 0.1 wt% (2.4 mM), [Phos-MecycC_5] = 0.4 wt% (6.5 mM), [GalNAc-cycC_6] = 0.2 wt% (3.1 mM), [Agarose] = 0.5 wt%, [DBS-COOH] = 0.2 wt% (4.5 mM), [glucono-δ-lactone] = 44.9 mM (for DBS-COOH), solvent: 100 mM MES pH 7.0 (except

for DBS-COOH) or water (for DBS-COOH), rt.

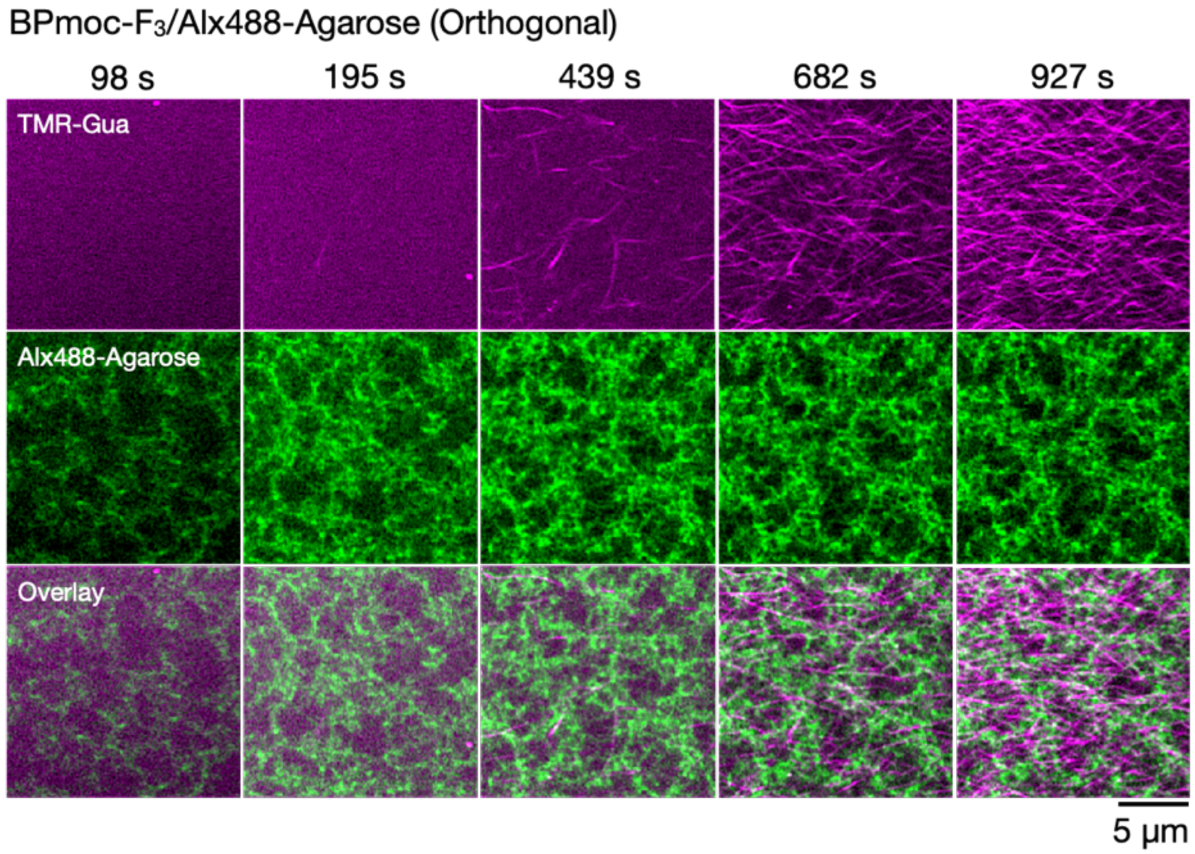

Supplementary Figure 17. Time-lapse CLSM imaging of the BPmoc-F_3/Alx488-Agarose composite hydrogel. Top: supramolecular fiber network (magenta), middle: agarose network (green), bottom: the merged images. Conditions: [BPmoc-F_3] = 0.1 wt% (1.6 mM), [TMR-Gua] = 14 µM, [Alx488-Agarose] = 0.5 wt%, solvent: 100 mM MES pH 7.0, scale bar: 5 µm, rt.

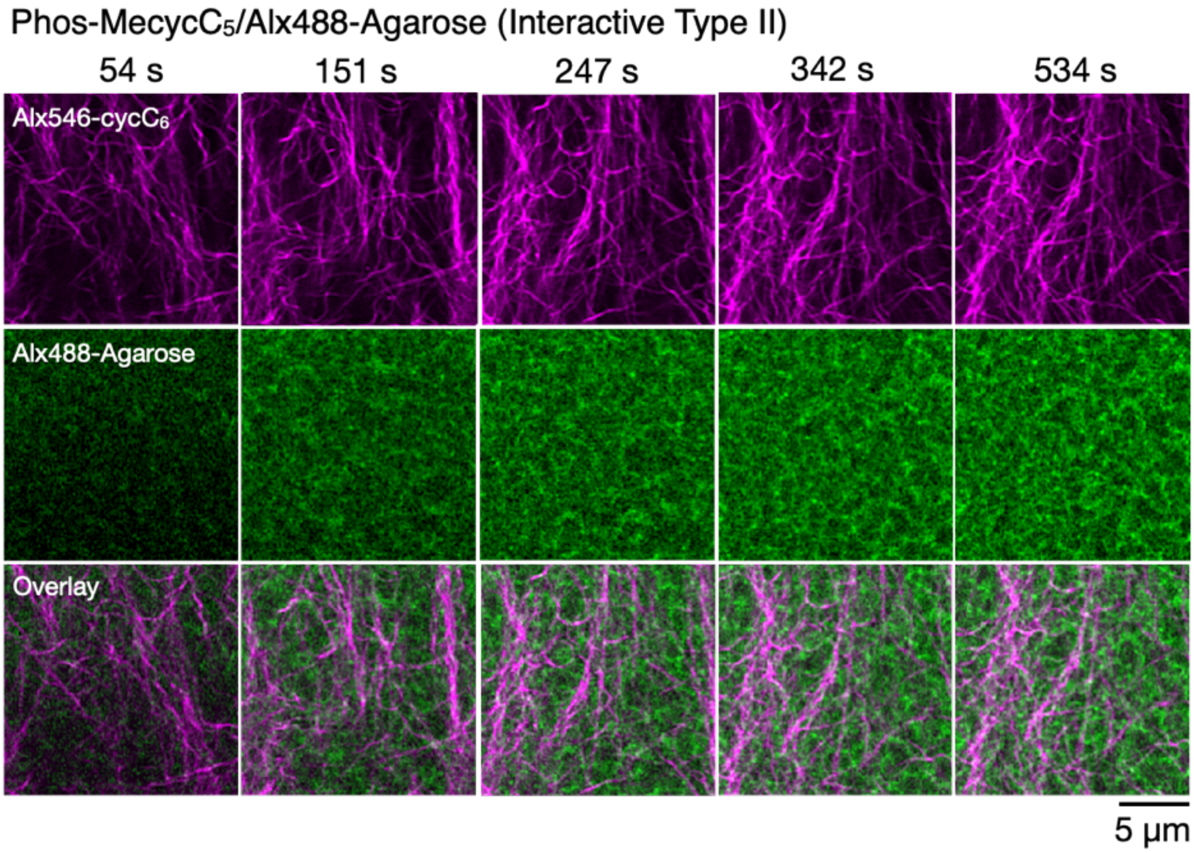

Supplementary Figure 18. Time-lapse CLSM imaging of the Phos-MecycC$_5$/Alx488-Agarose composite hydrogel. Top: supramolecular fiber network (magenta), middle: agarose network (green), bottom: the merged images. Conditions: [Phos-MecycC$_5$] = 0.4 wt% (6.5 mM), [Alx546-cycC$_6$] = 4 μM, [Alx488-Agarose] = 0.5 wt%, solvent: 100 mM MES pH 7.0, scale bar: 5 μm, rt.

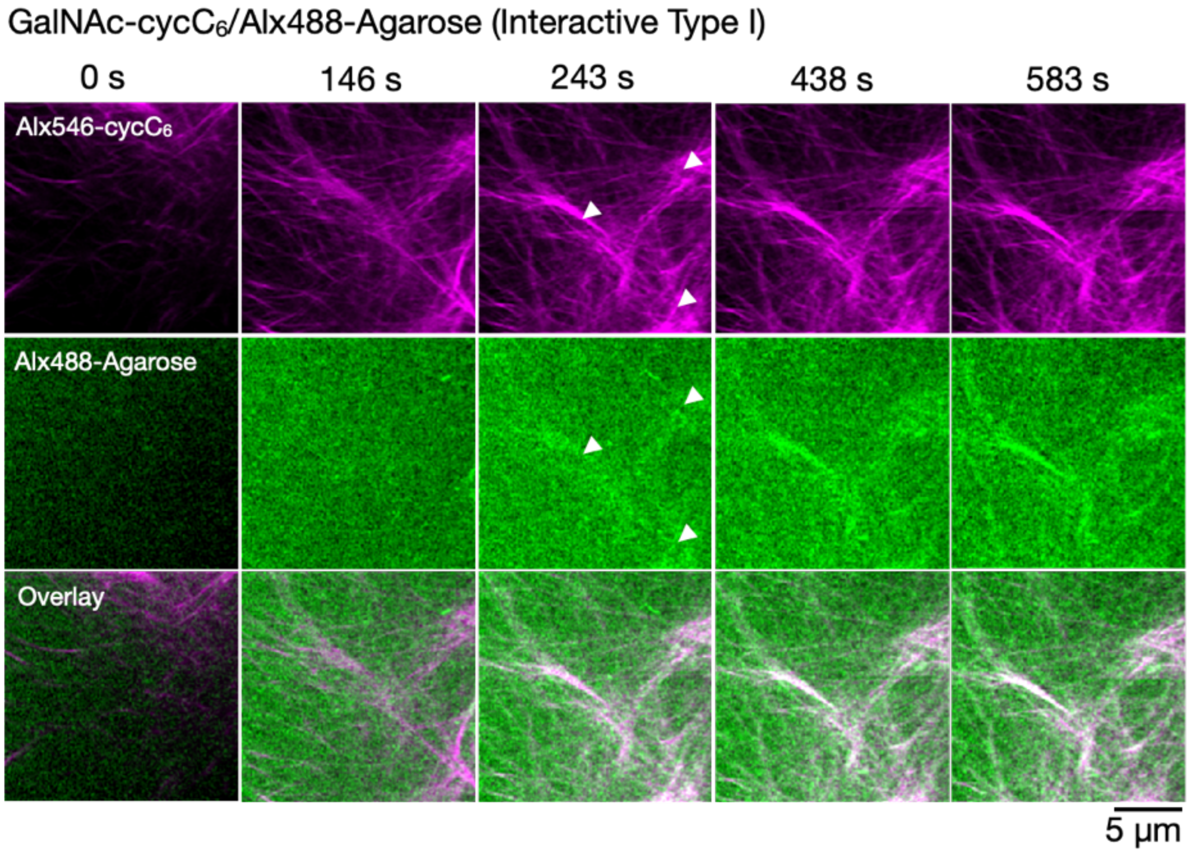

Supplementary Figure 19. Time-lapse CLSM imaging of the GalNAc-cycC_6/Alx488-Agarose composite hydrogel. Top: supramolecular fiber network (magenta), middle: agarose network (green), bottom: the merged images. The white arrow heads indicate the overlapped sites of each network. Conditions: [GalNAc-cycC_6] = 0.3 wt% (4.6 mM), [Alx546-cycC_6] = 4 μM, [Alx488-Agarose] = 0.5 wt%, solvent: 100 mM MES pH 7.0, scale bar: 5 μm, rt.

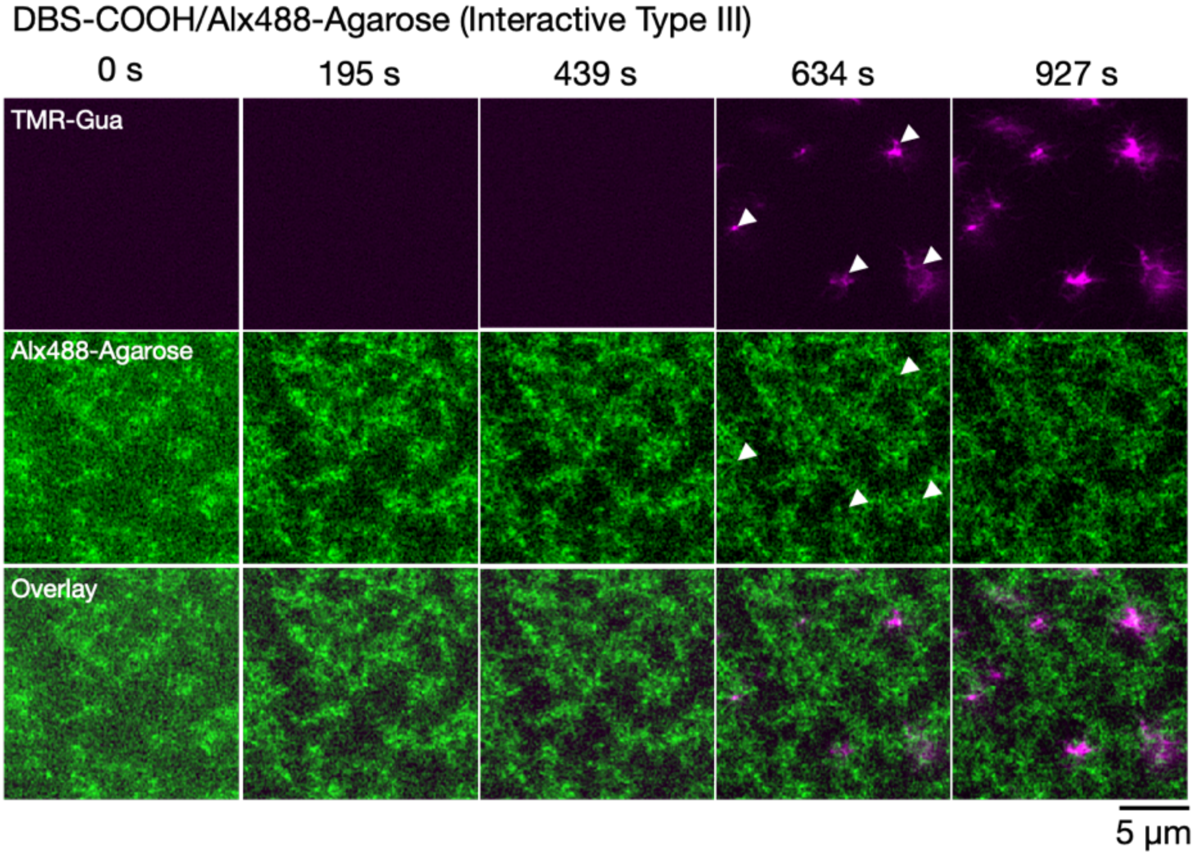

Supplementary Figure 20. Time-lapse CLSM imaging of the DBS-COOH/Alx488-Agarose composite hydrogel. Top: supramolecular fiber network (magenta), middle: agarose network (green), bottom: the merged images. The white arrow heads indicate the overlapped sites of each network. Conditions: [DBS-COOH] = 0.2 wt% (4.5 mM), [TMR-Gua] = 14 μM, [Alx488-Agarose] = 0.5 wt%, [glucono-δ-lactone] = 44.9 mM, solvent: water, scale bar: 5 μm, rt.

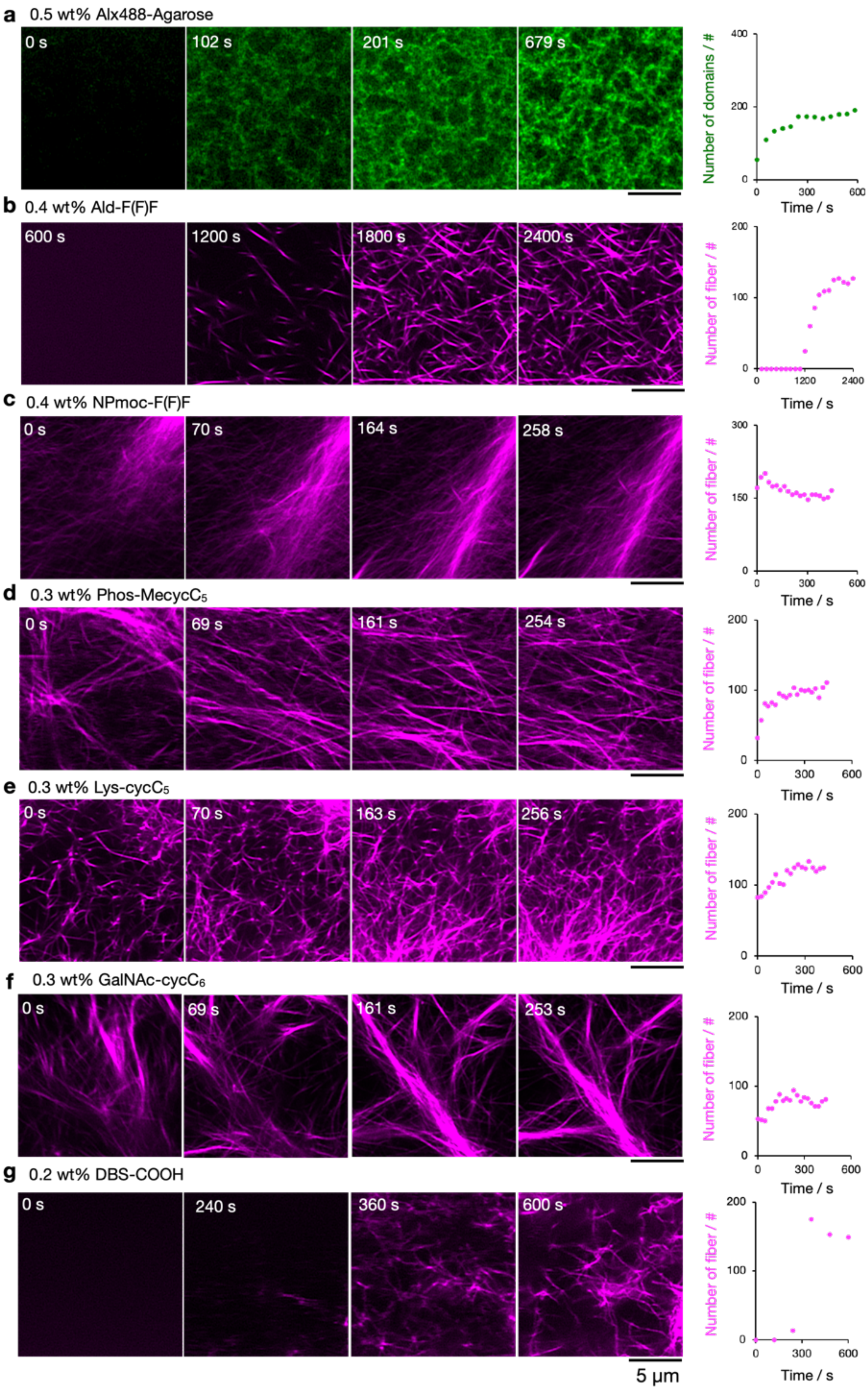
a 0.5 wt% Alx488-Agarose
0 s
102 s
201 s
679 s
Number of domains / #
Time / s
b 0.4 wt% Ald-F(F)F
600 s
1200 s
1800 s
2400 s
Number of fiber / #
Time / s
c 0.4 wt% NPmoc-F(F)F
0 s
70 s
164 s
258 s
Number of fiber / #
Time / s
d 0.3 wt% Phos-MecycC5
0 s
69 s
161 s
254 s
Number of fiber / #
Time / s
e 0.3 wt% Lys-cycC5
0 s
70 s
163 s
256 s
Number of fiber / #
Time / s
f 0.3 wt% GalNAc-cycC6
0 s
69 s
161 s
253 s
Number of fiber / #
Time / s
g 0.2 wt% DBS-COOH
0 s
240 s
360 s
600 s
5 μm
Number of fiber / #
Time / s

Supplementary Figure 21. Time-lapse CLSM imaging of the formation of the single-component network. (**a**) Alx488-Agarose, (**b**) Ald-F(F)F, (**c**) NPmoc-F(F)F, (**d**) Phos-MecycC$_5$, (**e**) Lys-cycC$_5$, (**f**) GalNAc-cycC$_6$, (**g**) DBS-COOH. The quantitative analyses of each network formation are shown on the right side of the images. The number of the nanofibers and the agarose domains in each time frame were counted. The time-lapse imaging of the single-component BPmoc-F$_3$ was already reported in our previous literature.[9] Conditions: [NPmoc-F(F)F] = 0.4 wt% (7.0 mM), [Ald-F(F)F] = 0.4 wt% (8.6 mM), [Phos-MecycC$_5$] = 0.4 wt% (6.5 mM), [Lys-cycC$_5$] = 0.3 wt% (5.9 mM), [GalNAc-cycC$_6$] = 0.3 wt% (4.6 mM), [DBS-COOH] = 0.2 wt% (4.5 mM), [TMR-Gua] = 14 μM, [Alx546-cycC$_6$] = 4 μM, [Alx488-Agarose] = 0.5 wt%, [glucono-δ-lactone] = 44.9 mM (**g**), solvent: 100 mM MES pH 7.0 (except for **g**) or water (**g**), scale bar: 5 μm, rt.

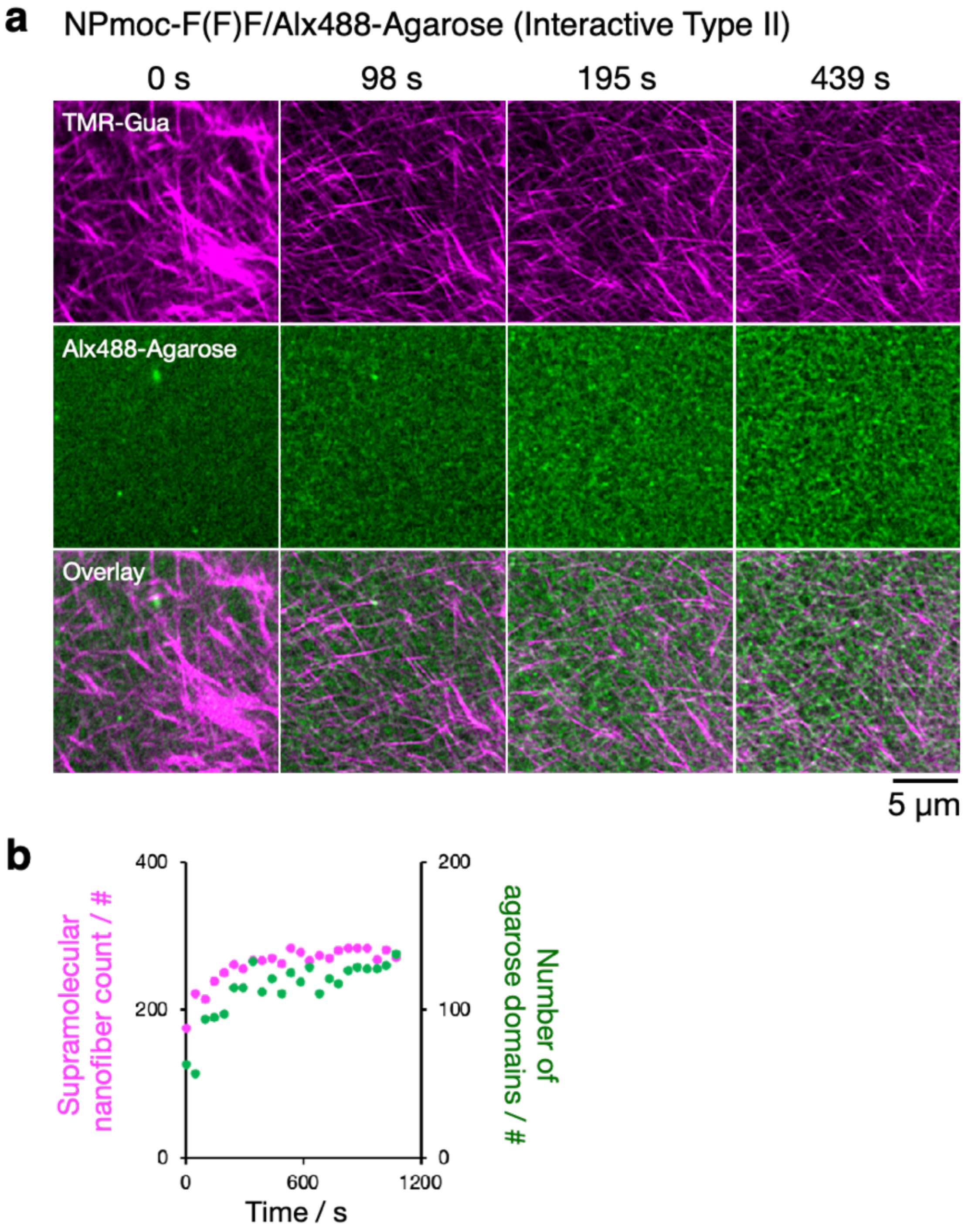

Supplementary Figure 22. **Time-lapse CLSM imaging of the NPmoc-F(F)F/Alx488-Agarose composite hydrogel.** (**a**) Time-course images. Top: supramolecular fiber network (magenta), middle: agarose network (green), bottom: the merged images. (**b**) Quantitative analysis of each network formation. The number of the nanofibers and the agarose domains in each time frame were counted. FL intensity: fluorescence intensity. Conditions: [NPmoc-F(F)F] = 0.4 wt% (7.9 mM), [TMR-Gua] = 14 μM, [Alx488-Agarose] = 0.5 wt%, solvent: 100 mM MES pH 7.0, scale bar: 5 μm, rt.

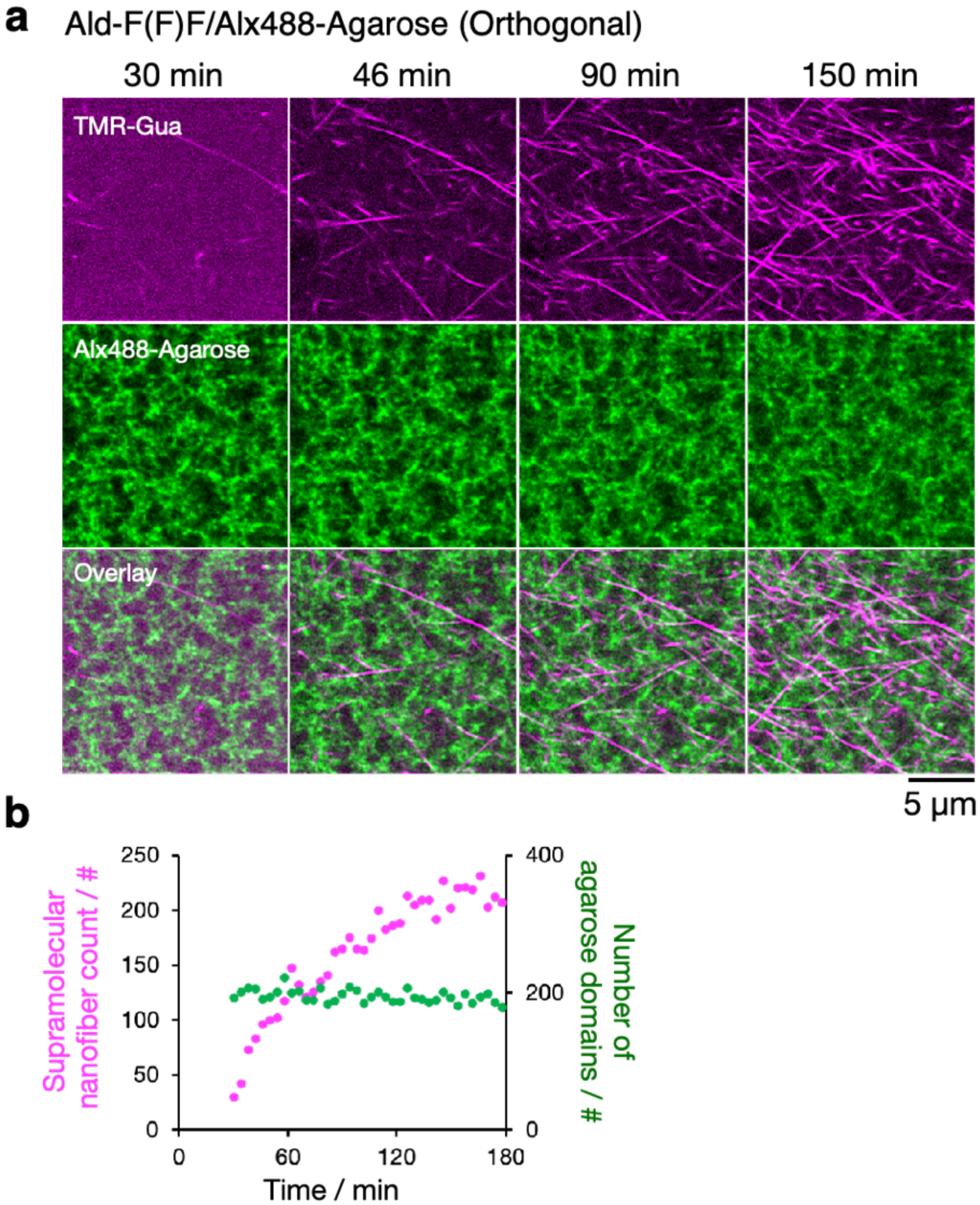

Supplementary Figure 23. Time-lapse CLSM imaging of the Ald-F(F)F/Alx488-Agarose composite hydrogel. (**a**) Time-course images. Top: supramolecular fiber network (magenta), middle: agarose network (green), bottom: the merged images. (**b**) Quantitative analysis of each network formation. The number of the nanofibers and the agarose domains in each time frame were counted. FL intensity: fluorescence intensity. Conditions: [Ald-F(F)F] = 0.4 wt% (8.6 mM), [TMR-Gua] = 14 μM, [Alx488-Agarose] = 0.5 wt%, solvent: 100 mM MES pH 7.0, scale bar: 5 μm, rt.

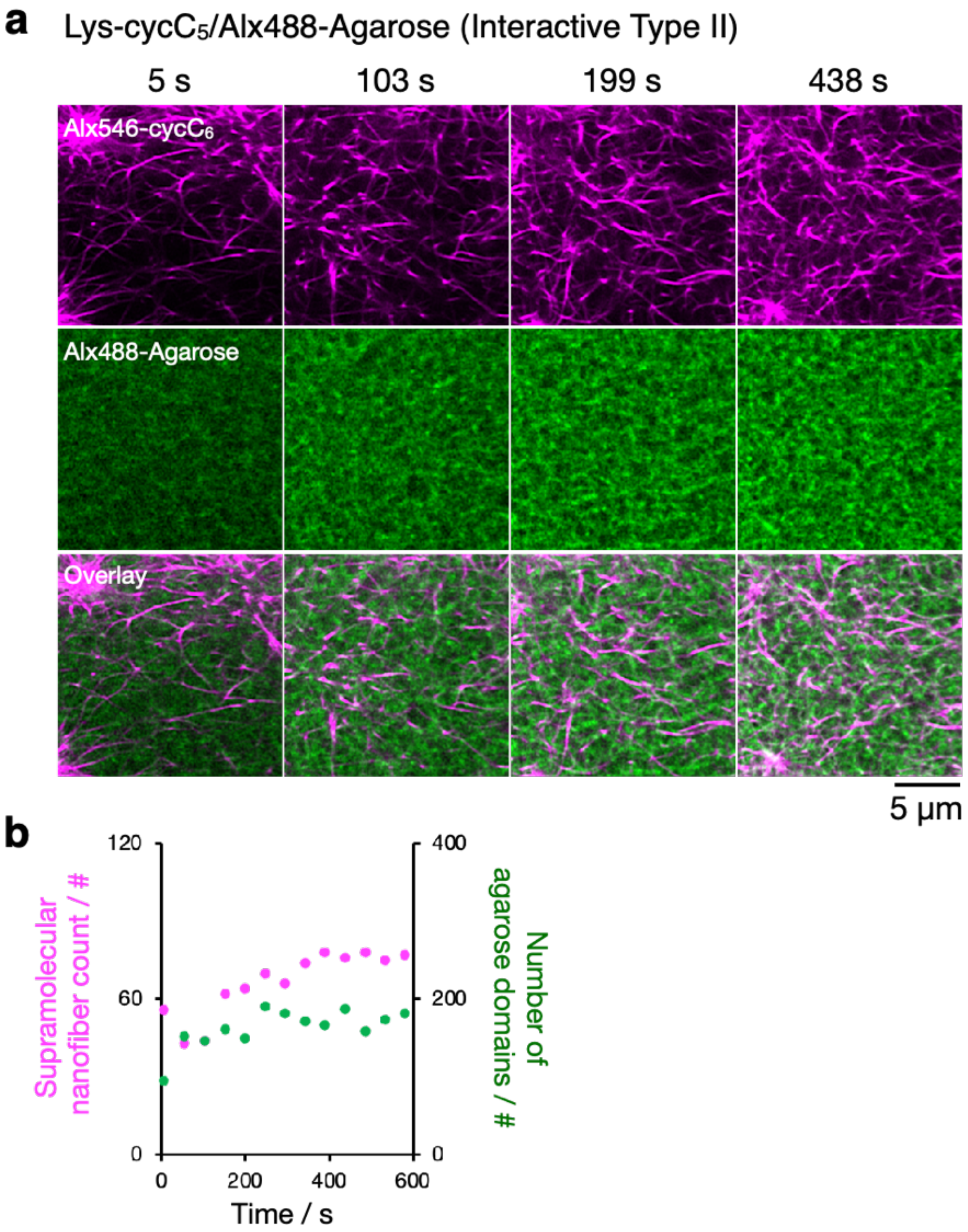

Supplementary Figure 24. Time-lapse CLSM imaging of the Lys-cycC$_5$/Alx488-Agarose composite hydrogel. (**a**) Time-course images. Top: supramolecular fiber network (magenta), middle: agarose network (green), bottom: the merged images. (**b**) Quantitative analysis of each network formation. The number of the nanofibers and the agarose domains in each time frame were counted. FL intensity: fluorescence intensity. Conditions: [Lys-cycC$_5$] = 0.3 wt% (5.9 mM), [Alx546-cycC$_6$] = 4 μM, [Alx488-Agarose] = 0.5 wt%, solvent: 100 mM MES pH 7.0, scale bar: 5 μm, rt.

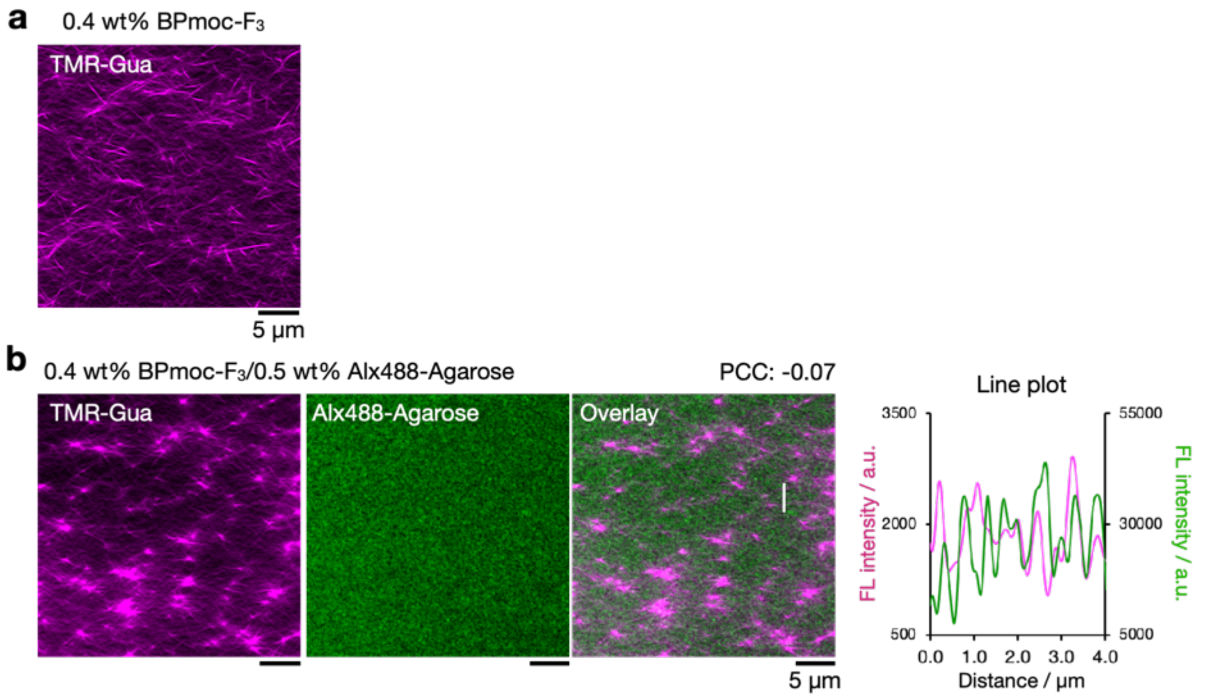

Supplementary Figure 25. CLSM imaging of the BPmoc-F_3/Alx488-Agarose hydrogel at higher BPmoc-F_3 concentration. (**a**) CLSM image of single component 0.4 wt% BPmoc-F_3. (**b**) CLSM images of 0.4 wt% BPmoc-F_3/0.5 wt% Alx488-Agarose. Magenta: supramolecular network, green: agarose. The right figure was line plot analysis along the white line shown in the merged image. The network was classifed as the interactive network type II. PCC: Pearson's correlation coefficient. FL intensity: Fluorescence intensity, a.u.: arbitrary units. Conditions: [BPmoc-F_3] = 0.4 wt% (6.4 mM), [Alx488-Agarose] = 0.5 wt%, [TMR-Gua] = 14 μM, solvent: 100 mM MES pH 7.0, scale bar: 5 μm, rt.

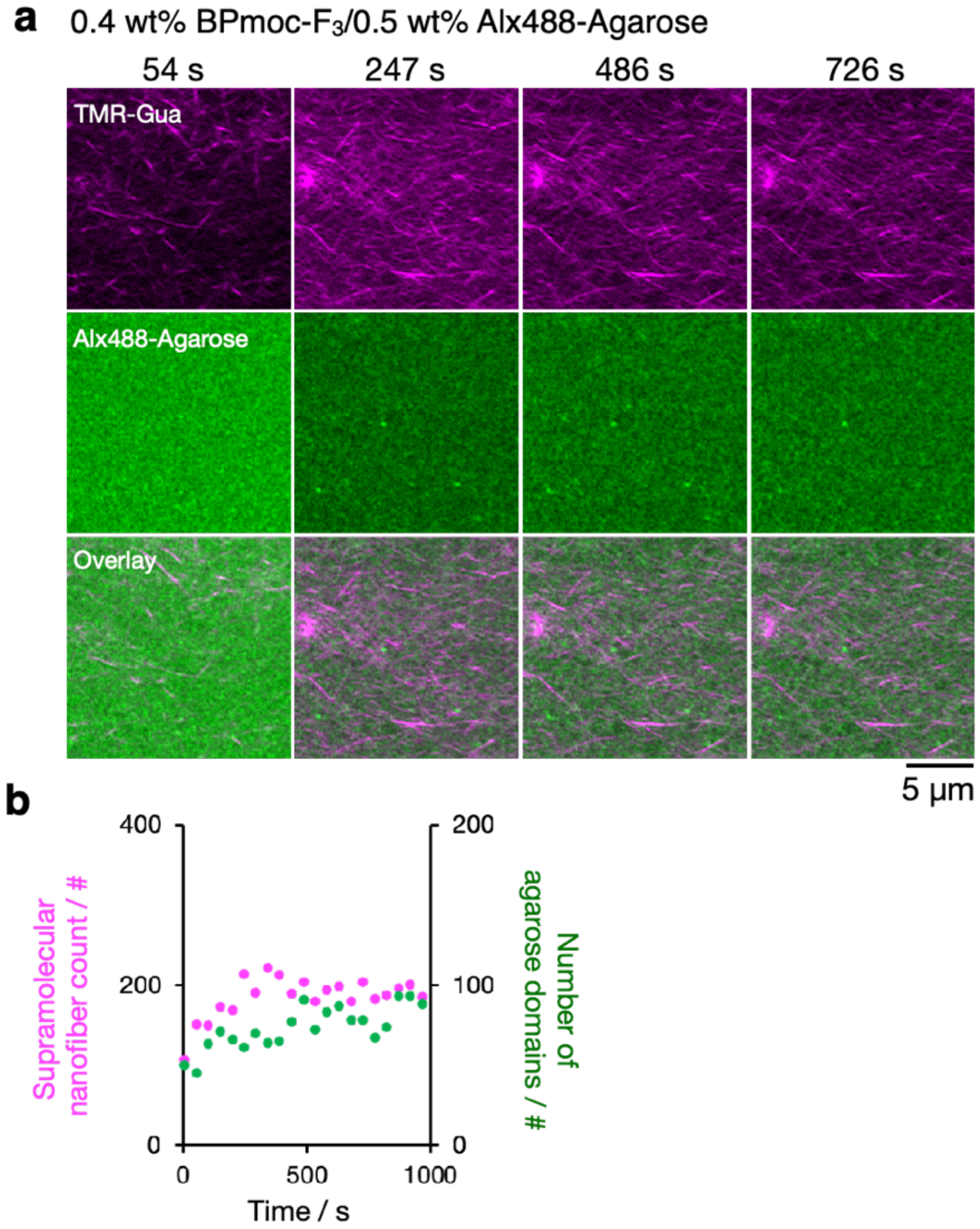

Supplementary Figure 26. Time-lapse CLSM imaging of the formation process of the BPmoc-F$_3$/Alx488-Agarose hydrogel at higher BPmoc-F$_3$ concentration. (**a**) Time-course images. Top: supramolecular fiber network (magenta), middle: agarose network (green), bottom: the merged images. (**b**) Quantitative analysis of each network formation. The number of the nanofibers and the agarose domains in each time frame were counted. FL intensity: fluorescence intensity. Conditions: [BPmoc-F$_3$] = 0.4 wt% (6.4 mM), [Alx488-Agarose] = 0.5 wt%, [TMR-Gua] = 14 µM, solvent: 100 mM MES pH 7.0, rt, scale bar: 5 µm, rt.

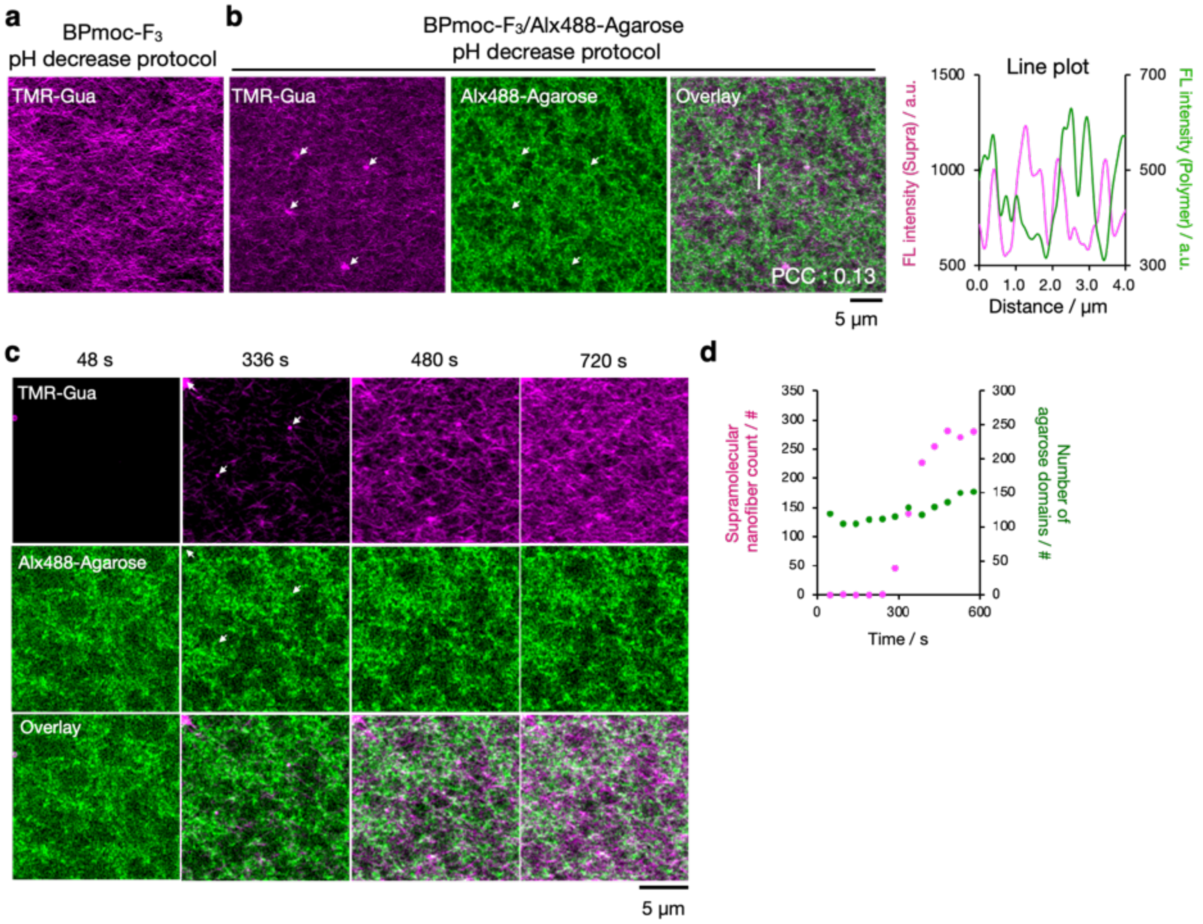

Supplementary Figure 27. CLSM imaging of the BPmoc-F$_3$/Alx488-Agarose hydrogel prepared by a pH decrease protocol. (**a**) CLSM image of single component 0.2 wt% BPmoc-F$_3$ with glucono-δ-lactone. (**b**) CLSM images of 0.2 wt% BPmoc-F$_3$/0.5 wt% Alx488-Agarose with glucono-δ-lactone. Magenta: TMR-Gua, green: Alx488-Agarose. The right figure was the line plot analysis along the white line shown in the merged image. FL intensity: fluorescence intensity, Supra: supramolecular network, a.u.: arbitrary units. (**c**) Time-lapse CLSM imaging of the formation process of the BPmoc-F$_3$/Alx488-Agarose hydrogel during pH decrease. (**d**) Quantitative analysis of each network formation. PCC: Pearson's correlation coefficient. FL intensity: Fluorescence intensity. Conditions: [BPmoc-F$_3$] = 0.2 wt% (3.2 mM), [Alx488-Agarose] = 0.5 wt%, [TMR-Gua] = 14 μM, [glucono-δ-lactone] = 4.49 mM, solvent: water, scale bar: 5 μm, rt.

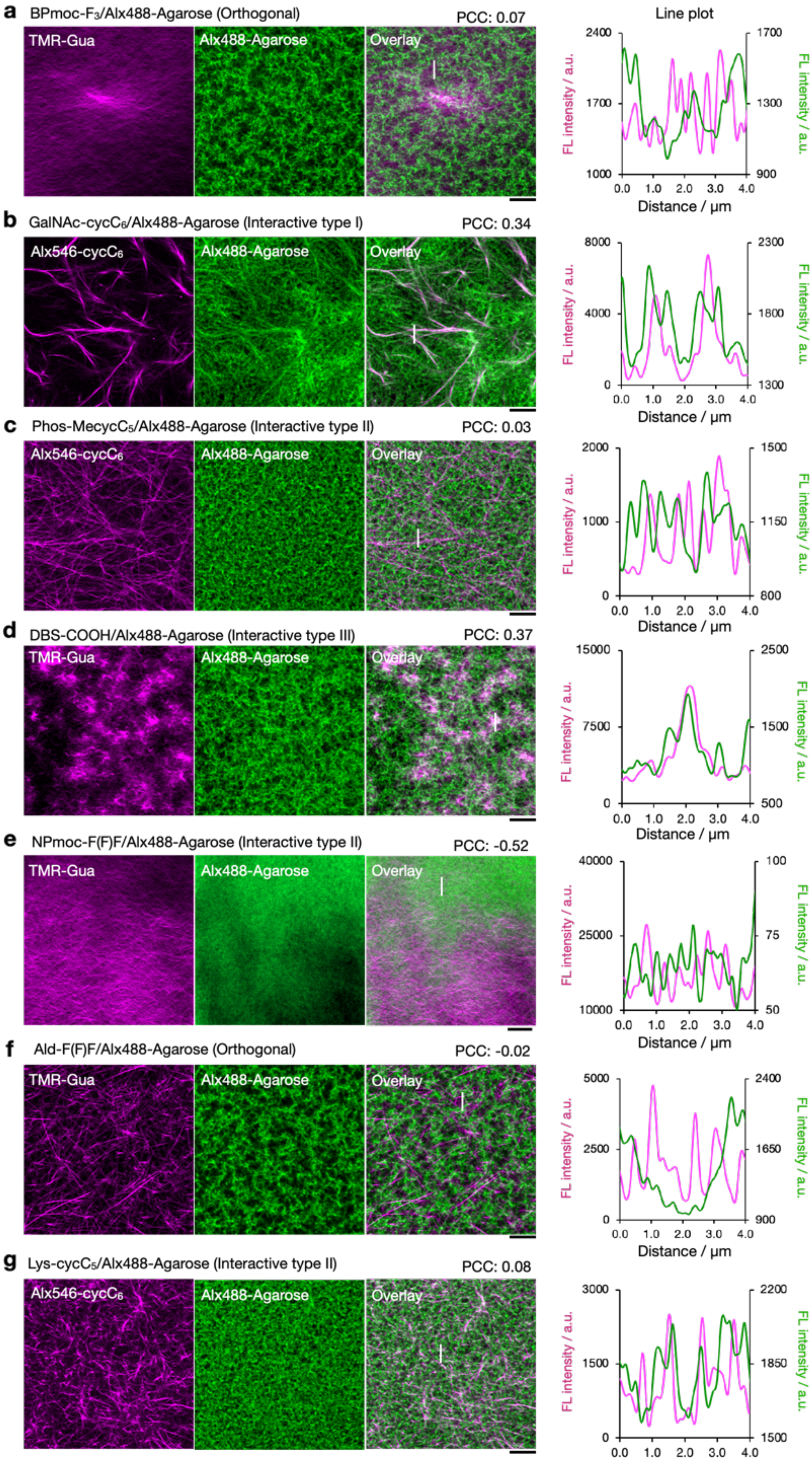

a BPmoc-F3/Alx488-Agarose (Orthogonal)
PCC: 0.07
TMR-Gua
Alx488-Agarose
Overlay
Line plot
b GalNAc-cycC6/Alx488-Agarose (Interactive type I)
PCC: 0.34
Alx546-cycC6
c Phos-MecycC5/Alx488-Agarose (Interactive type II)
PCC: 0.03
d DBS-COOH/Alx488-Agarose (Interactive type III)
PCC: 0.37
e NPmoc-F(F)F/Alx488-Agarose (Interactive type II)
PCC: -0.52
f Ald-F(F)F/Alx488-Agarose (Orthogonal)
PCC: -0.02
g Lys-cycC5/Alx488-Agarose (Interactive type II)
PCC: 0.08
FL intensity / a.u.
Distance / µm

Supplementary Figure 28. High-resolution CLSM images of the network structure after aging for 48 h. (**a**) BPmoc-F_3/Alx488-Agarose, (**b**) GalNAc-cycC_6/Alx488-Agarose, (**c**) Phos-MecycC_5/Alx488-Agarose, (**d**) DBS-COOH/Alx488-Agarose, (**e**) NPmoc-F(F)F/Alx488-Agarose, (**f**) Ald-F(F)F/Alx488-Agarose, and (**g**) Lys-cycC_5/Alx488-Agarose after 48 h incubation. For these composite hydrogels, the type of the network pattern kept unchanged. The right figures are line plot analyses along the white line shown on the merged images. PCC: Pearson's correlation coefficient. FL intensity: fluorescence intensity, a.u.: arbitrary units. Conditions: [BPmoc-F_3] = 0.1 wt% (1.6 mM), [NPmoc-F(F)F] = 0.4 wt% (7.0 mM), [Ald-F(F)F] = 0.4 wt% (8.6 mM), [Phos-MecycC_5] = 0.4 wt% (6.5 mM), [Lys-cycC_5] = 0.3 wt% (5.9 mM), [GalNAc-cycC_6] = 0.3 wt% (4.6 mM), [DBS-COOH] = 0.2 wt% (4.5 mM), [TMR-Gua] = 14 µM, [Alx546-cycC_6] = 4 µM, [Alx488-Agarose] = 0.5 wt%, [glucono-δ-lactone] = 44.9 mM (**d**), solvent: 100 mM MES pH 7.0 (except for **d**) or water (**d**), scale bar: 5 µm, rt.

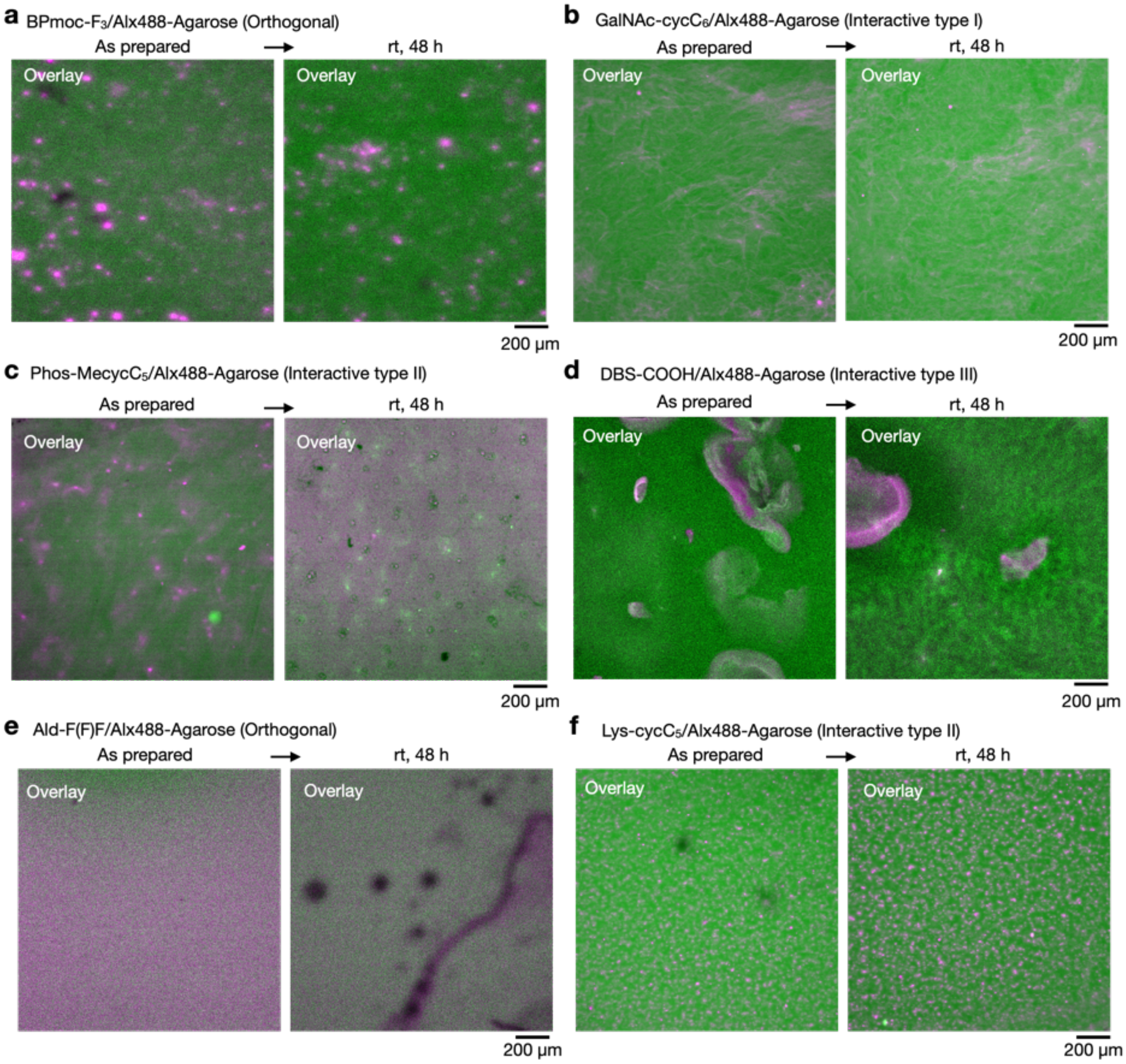

Supplementary Figure 29. Low-magnification CLSM images of composite hydrogels before and after aging for 48 h. (**a**) BPmoc-F_3/Alx488-Agarose, (**b**) GalNAc-cycC_6/Alx488-Agarose, (**c**) Phos-MecycC_5/Alx488-Agarose, (**d**) DBS-COOH/Alx488-Agarose, (**e**) Ald-F(F)F/Alx488-Agarose, and (**f**) Lys-cycC_5/Alx488-Agarose (left) before and (right) after 48 h incubation. No significant changes in network structures were observed in these composite hydrogels. Different fields of view were observed before and after incubation. Conditions: [BPmoc-F_3] = 0.1 wt% (1.6 mM), [Ald-F(F)F] = 0.4 wt% (8.6 mM), [Phos-MecycC_5] = 0.4 wt% (6.5 mM), [Lys-cycC_5] = 0.3 wt% (5.9 mM), [GalNAc-cycC_6] = 0.3 wt% (4.6 mM), [DBS-COOH] = 0.2 wt% (4.5 mM), [TMR-Gua] = 14 µM, [Alx546-cycC_6] = 4 µM, [Alx488-Agarose] = 0.5 wt%, [glucono-δ-lactone] = 44.9 mM (**d**), solvent: 100 mM MES pH 7.0 (except for **d**) or water (**d**), scale bar: 200 µm, rt.

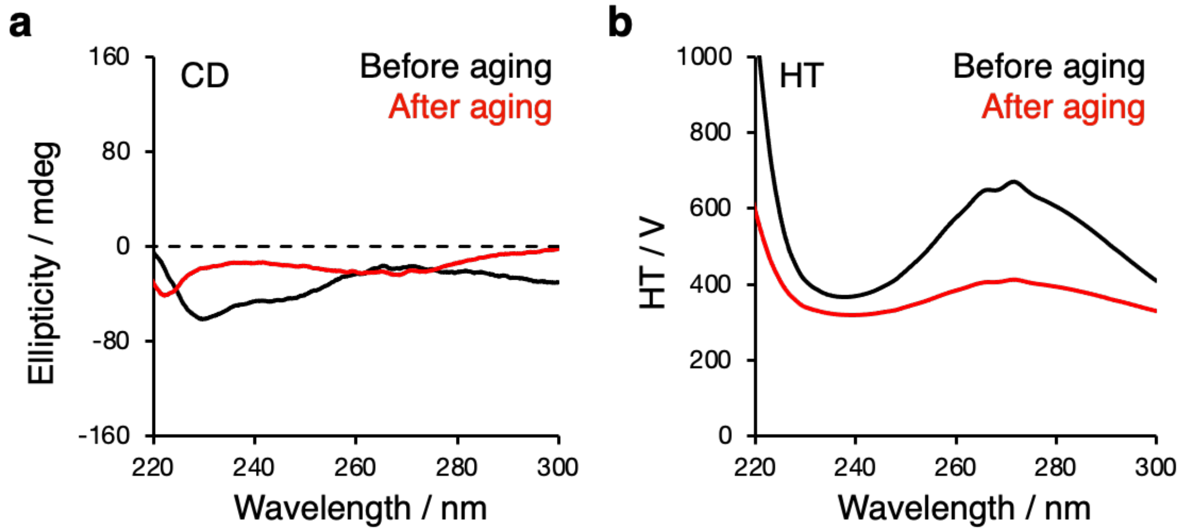

Supplementary Figure 30. Time-dependent change of the self-assembly state of the NPmoc-F(F)F/Agarose. (**a**) CD spectra of the NPmoc-F(F)F/Agarose composite hydrogel before and after aging. (**b**) HT voltage data of the NPmoc-F(F)F/Agarose composite hydrogel before and after aging. Agarose does not show any Cotton peaks under the current experimental condition. Conditions: [NPmoc-F(F)F] = 0.4 wt% (8.6 mM), [Agarose] = 0.5 wt%, 100 mM MES buffer (pH 7.0).

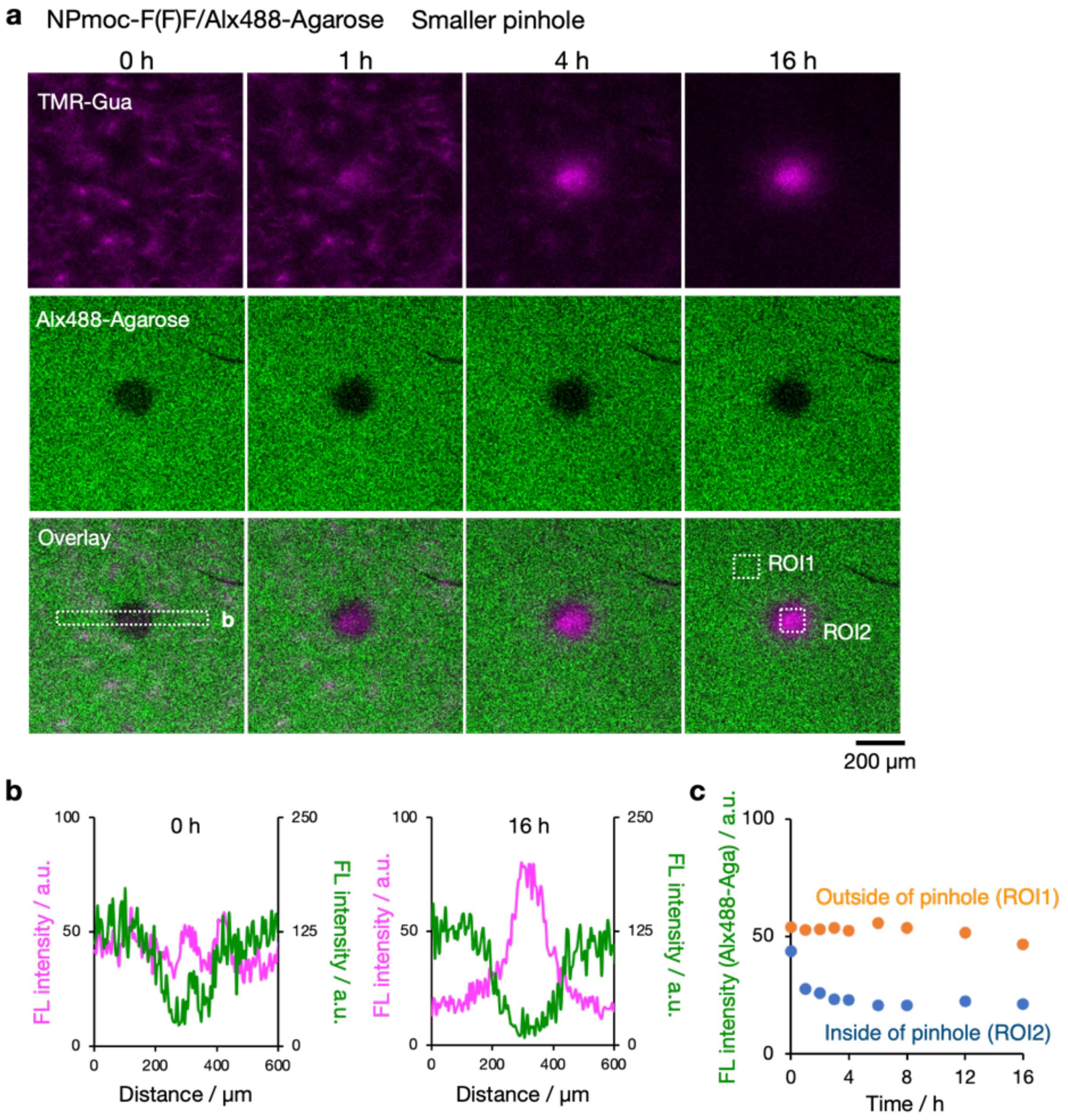

Supplementary Figure 31. Time-lapse CLSM imaging of the NPmoc-F(F)F/Alx488-Agarose composite hydrogel after puncture with a smaller pinhole. (**a**) Low-magnification CLSM images of NPmoc-F(F)F/Alx488-Agarose in the presence of a smaller pinhole (*ca.* 100 μm). Magenta: supramolecular network, green: agarose. (**b**) Line plot analyses of the mean FL (fluorescence) intensity at (left) 0 h and (right) 16 h after the puncture at each coordinate along horizontal axis of the white square as shown in the overlay image at 0 h. FL intensity: fluorescence intensity, a.u.: arbitrary units. (**c**) Time-course changes of FL intensity of the Alx488-Agarose in the ROIs. Orange: ROI1 (outside of the pinhole), blue: ROI2 (inside of the pinhole). Conditions: [NPmoc-F(F)F] = 0.4 wt% (7.0 mM), [Alx488-Agarose] = 0.5 wt%, [TMR-Gua] = 14 μM, solvent: 100 mM MES pH 7.0, scale bar: 200 μm, rt.

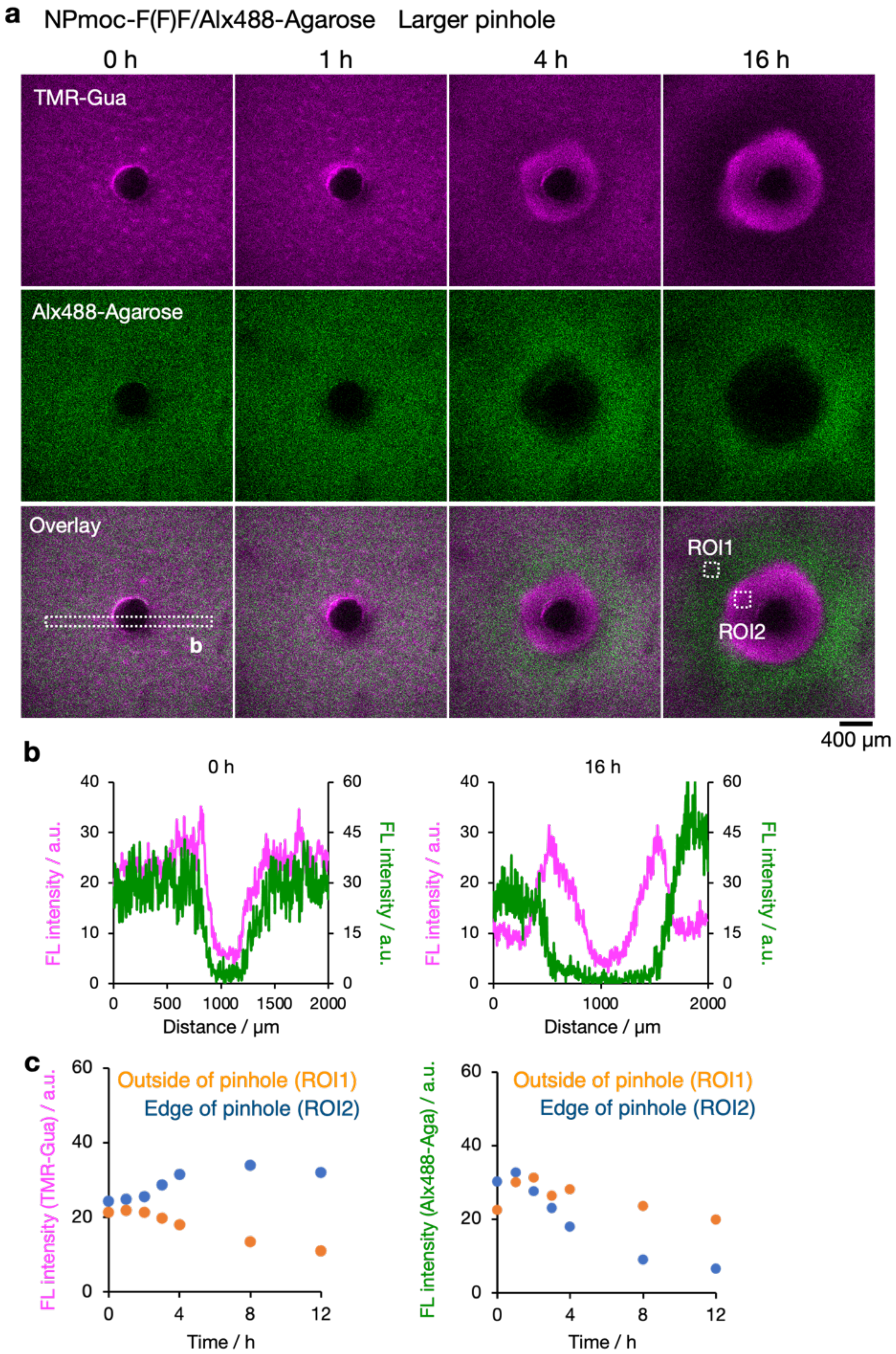
a
NPmoc-F(F)F/Alx488-Agarose Larger pinhole
0 h
1 h
4 h
16 h
TMR-Gua
Alx488-Agarose
Overlay
b
ROI1
ROI2
400 µm
b
0 h
16 h
FL intensity / a.u.
FL intensity / a.u.
Distance / µm
c
Outside of pinhole (ROI1)
Edge of pinhole (ROI2)
FL intensity (TMR-Gua) / a.u.
FL intensity (Alx488-Aga) / a.u.
Time / h

Supplementary Figure 32. Time-lapse CLSM imaging of the NPmoc-F(F)F/Alx488-Agarose composite hydrogel after puncture with a larger pinhole. (**a**) Low-magnification CLSM images of NPmoc-F(F)F/Alx488-Agarose in the presence of a larger pinhole (*ca*. 500 μm). Magenta: supramolecular network, green: agarose. (**b**) Line plot analyses of the mean FL (fluorescence) intensity at (left) 0 h and (right) 16 h after the puncture at each coordinate along horizontal axis of the white square as shown in the overlay image at 0 h. FL intensity: fluorescence intensity, a.u.: arbitrary units. (**c**) Time-couse changes of FL intensity of the (left) TMR-Gua or (Right) Alx488-Agarose in the ROIs. Orange: ROI1 (outside of the pinhole), blue: ROI2 (egde of the pinhole). Conditions: [NPmoc-F(F)F] = 0.4 wt% (7.0 mM), [Alx488-Agarose] = 0.5 wt%, [TMR-Gua] = 14 μM, solvent: 100 mM MES pH 7.0, scale bar: 400 μm, rt.

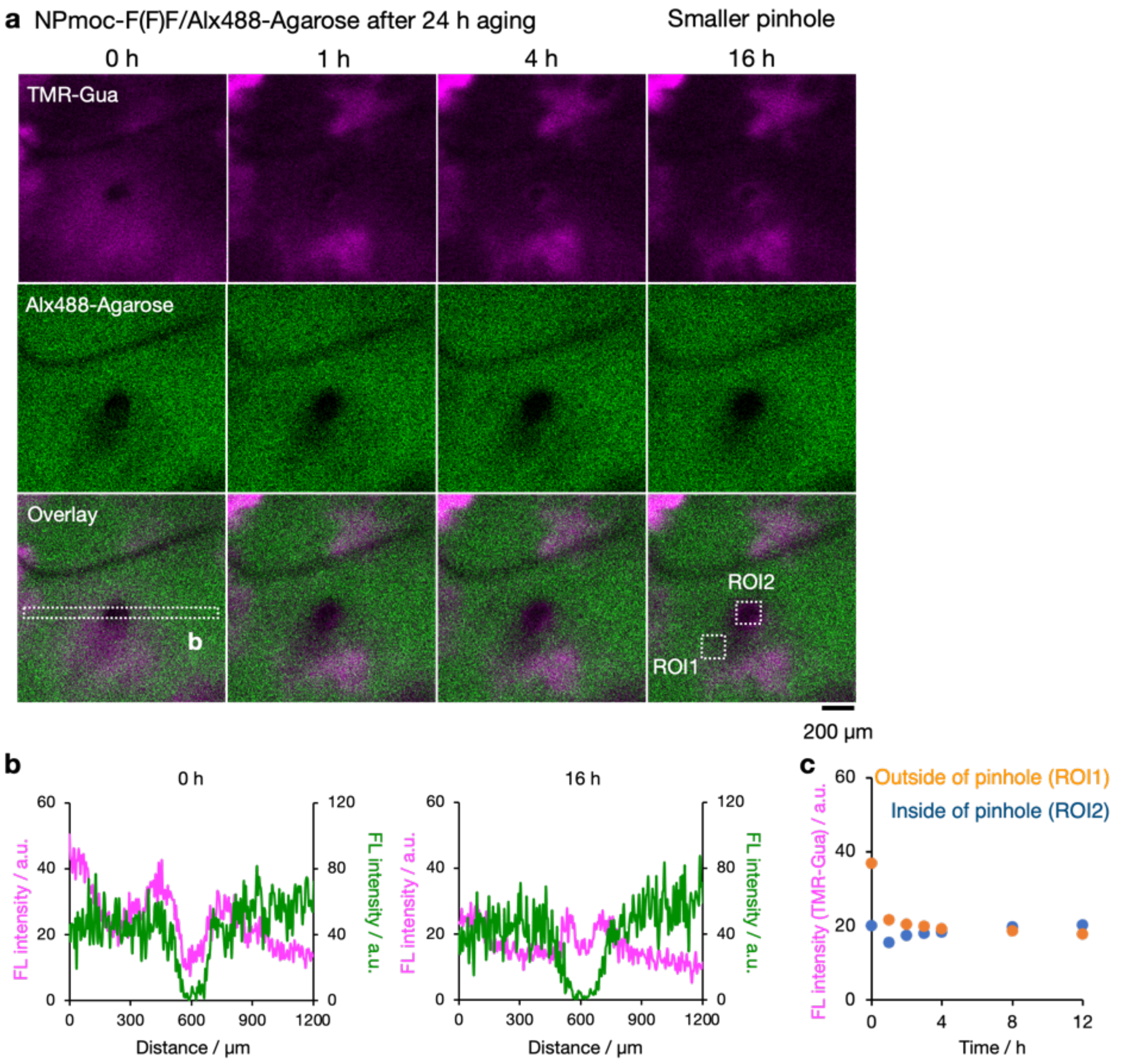

Supplementary Figure 33. Time-lapse CLSM imaging of the 24 h aged sample of the NPmoc-F(F)F/Alx488-Agarose composite hydrogel after puncture with a smaller needle. (**a**) Low-maginification CLSM images of the 24 h aged hydrogel of NPmoc-F(F)F/Alx488-Agarose in the presence of a smaller pinhole. Magenta: supramolecular network, green: agarose. (**b**) Line plot analyses of the mean FL (fluorescence) intensity at 0 h and 16 h after the puncture at each coordinate along horizontal axis of the white square as shown in the overlay image at 0 h. FL intensity: fluorescence intensity, a.u.: arbitrary units. (**c**) Time-course changes of FL intensity of the Alx488-Agarose in the ROIs. Orange: ROI1 (outside of the pinhole), blue: ROI2 (inside of the pinhole). Conditions: [NPmoc-F(F)F] = 0.4 wt% (7.0 mM), [Alx488-Agarose] = 0.5 wt%, [TMR-Gua] = 14 μM, solvent: 100 mM MES pH 7.0, scale bar: 200 μm, rt.

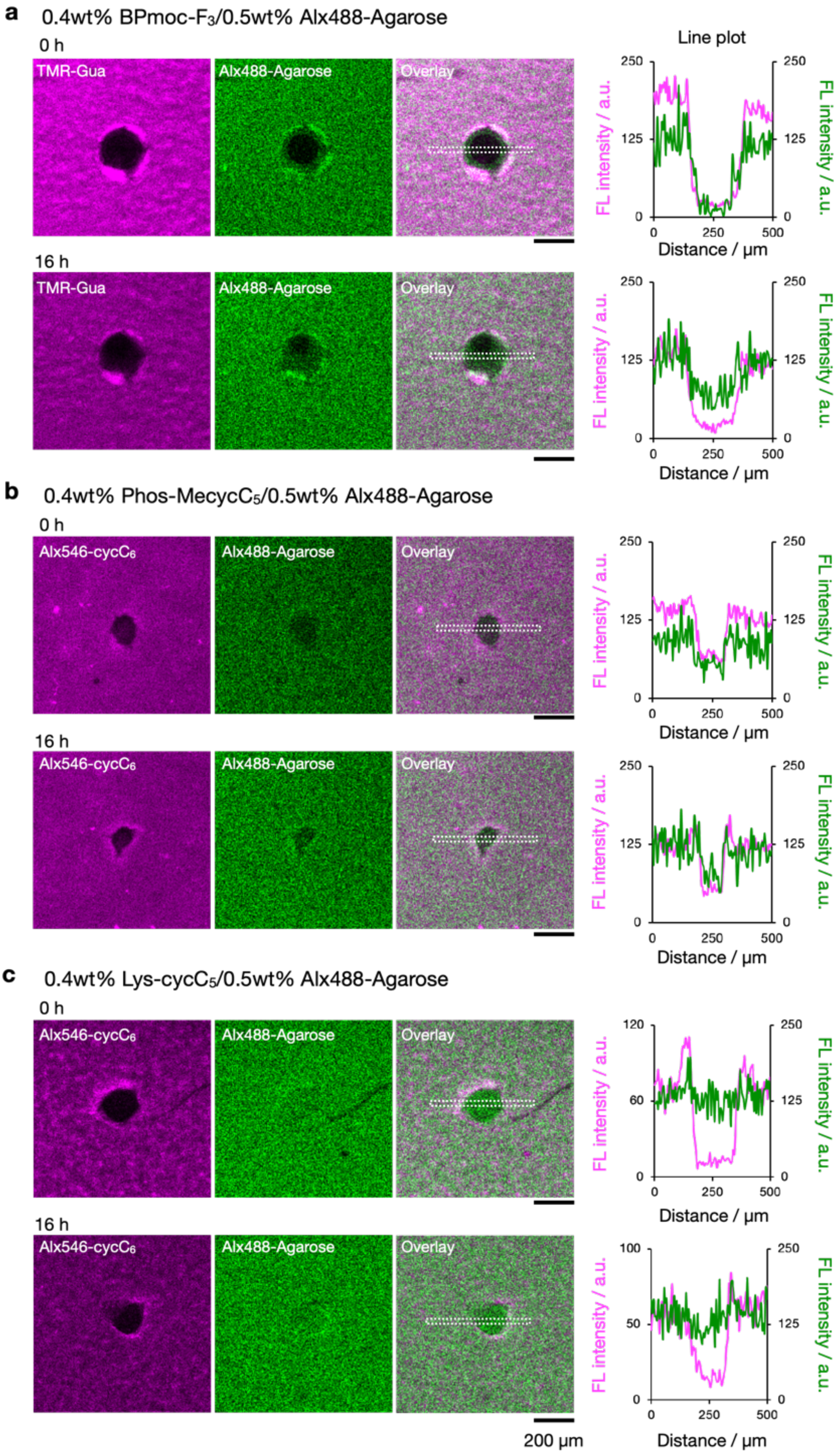
a
0.4wt% BPmoc-F3/0.5wt% Alx488-Agarose
0 h
TMR-Gua
Alx488-Agarose
Overlay
Line plot
16 h
FL intensity / a.u.
Distance / μm
b
0.4wt% Phos-MecycC5/0.5wt% Alx488-Agarose
Alx546-cycC6
c
0.4wt% Lys-cycC5/0.5wt% Alx488-Agarose
200 μm

Supplementary Figure 34. Time-lapse CLSM imaging of the composite hydrogel with interactive type II after puncture with a smaller needle. (**a**, **b**, **c**) Low-maginification CLSM images of the composite hydrogels in the presence of a smaller pinhole before and after aging. Magenta: supramolecular network, green: agarose. (**a**) 0.4 wt% BPmoc-F_3/0.5 wt% Alx488-Agarose, (**b**) 0.4 wt% Phos-MecycC_5/0.5 wt% Alx488-Agarose,, and (**c**) 0.4wt% Lys-cycC_5/0.5 wt% Alx488-Agarose. The right figures are line plot analyses of the mean FL (fluorescence) intensity at (left) 0 h and (right) 16 h after the puncture at each coordinate along the horizontal axis of the white square as shown in the overlay image. a.u.: arbitrary units. Conditions: [BPmoc-F_3] = 0.4 wt% (6.4 mM), [Phos-MecycC_5] = 0.4 wt% (6.5 mM), [Lys-cycC_5] = 0.4 wt% (5.9 mM), [Alx488-Agarose] = 0.5 wt%, [TMR-Gua] = 14 μM, solvent: 100 mM MES pH 7.0, scale bar: 200 μm, rt.

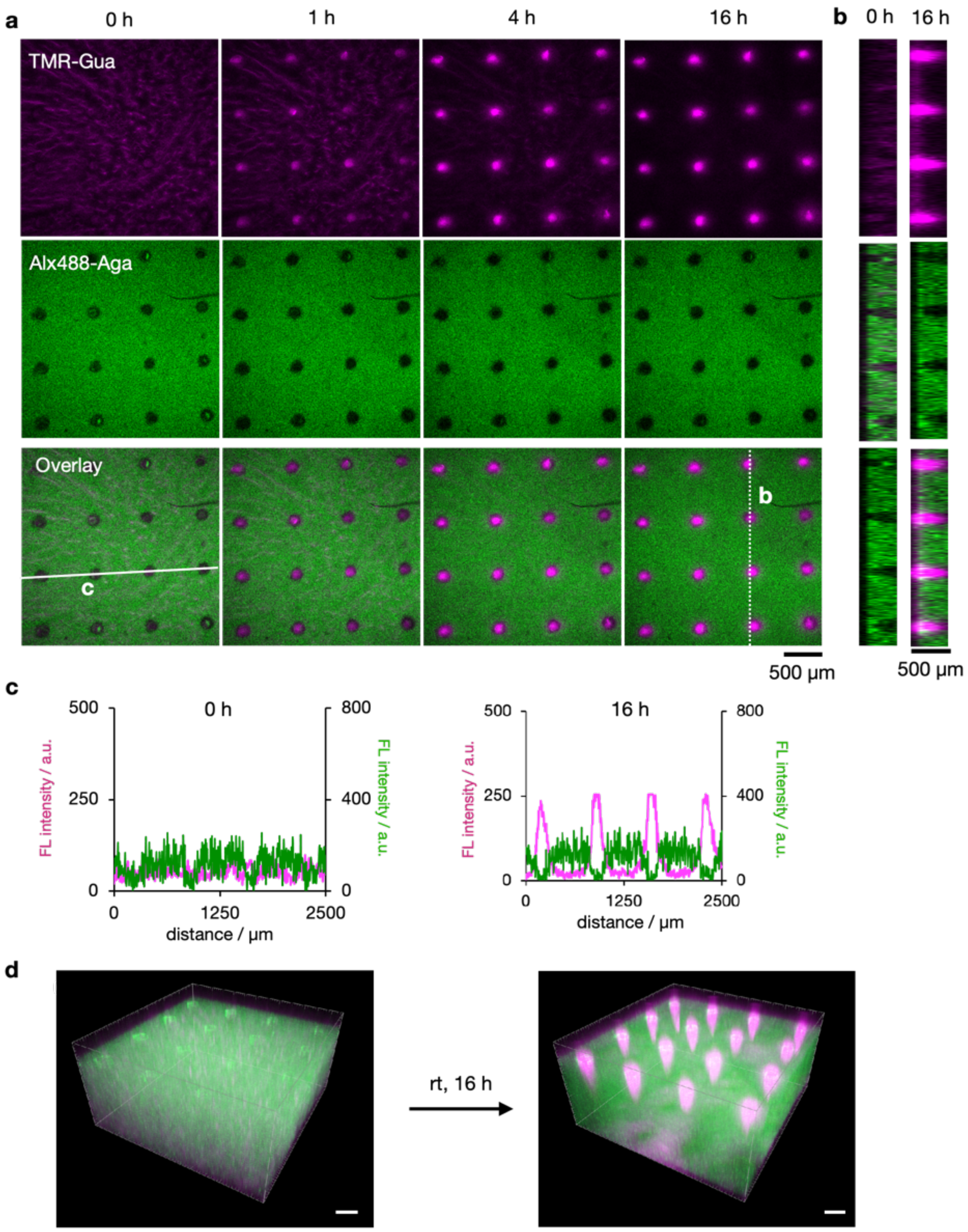

Supplementary Figure 35. Fracture-induced patterning of the NPmoc-F(F)F/Alx488-Agarose composite hydrogel after puncture with a dot–grid patterned microneedle stamp. (**a**) Time-lapse CLSM images of a dot–grid–pattern formation during incubation at rt after punctured with a stamp (shown in Figure 5f). (**b**) *yz* sectional views along the white dotted line in Supplementary Figure 35a. (**c**) Line plot analyses along the white lines shown in Supplementary Fig. 35a. Magenta: supramolecular network, green: agarose network. FL

intensity: Fluorescence intensity, a.u.: arbitrary units. (**d**) 3D images of the punctured hydrogel (left) before and (right) after incubation. Conditions: [NPmoc-F(F)F] = 0.4 wt% (7.0 mM), [Alx488-Agarose] = 0.5 wt%, [TMR-Gua] = 14 μM, solvent: 100 mM MES pH 7.0, rt, scale bar: 500 μm (**a**) and 300 μm (**d**).

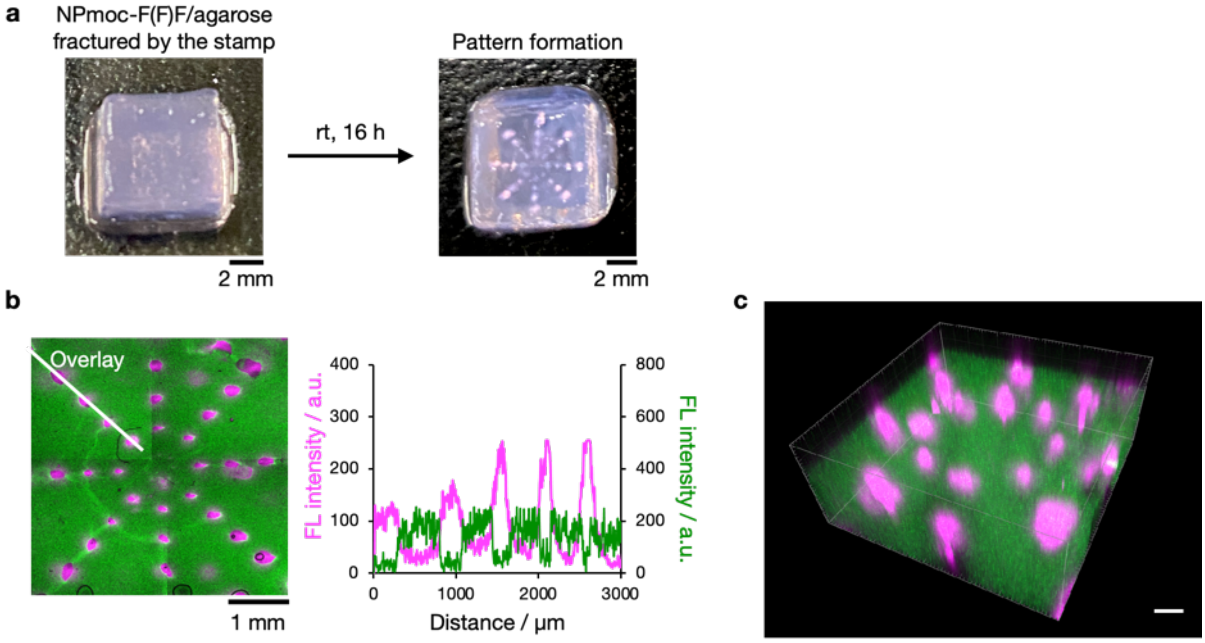

Supplementary Figure 36. Fracture-induced patterning of the NPmoc-F(F)F/Alx488-Agarose composite hydrogel after puncture with a concentric-patterned microneedles. (**a**) Photographs of the NPmoc-F(F)F/Alx488-Agarose composite hydrogel (left) before and (right) after the pucture with a concentric-patterned microneedle stamp (shown in Figure 5h). (**b**) (Left) CLSM image of the punctured hydrogel after incubation and (right) line plot analyses along the white lines shown in the CLSM image. Magenta: supramolecular network, green: agarose network. FL intensity: Fluorescence intensity, a.u.: arbitrary units. (**c**) 3D image of the patterned composite hydrogel. Conditions: [NPmoc-F(F)F] = 0.4 wt% (7.0 mM), [Alx488-Agarose] = 0.5 wt%, [TMR-Gua] = 14 µM, solvent: 100 mM MES pH 7.0, rt, scale: 2 mm (**a**), 1 mm (**b**), and 300 µm (**c**).

Supplementary Table

Supplementary Table 1. The average peak distance of each network pattern (*n* = 115). The data present the mean ± s.d.

	Peak distance / nm
BPmoc-F_3/Alx488-Agarose (Orthogonal)	200 ± 200
GalNAc-cycC_6/Alx488-Agarose (Interactive type I)	120 ± 110
Phos-MecycC_5/Alx488-Agarose (Interactive type II)	210 ± 170
DBS-COOH/Alx488-Agarose (Interactive type III)	150 ± 170

Supplementary Table 2. Summary of network classification

Supramolecular network	Polymer network	Classification
0.1 wt% BPmoc-F_3	0.5 wt% Agarose	Orthogonal
0.3 wt% GalNAc-cycC_6	0.5 wt% Agarose	Interactive I
0.4 wt% Phos-MecycC_5	0.5 wt% Agarose	Interactive II
0.2 wt% DBS-COOH	0.5 wt% Agarose	Interactive III
0.4 wt% NPmoc-F(F)F	0.5 wt% Agarose	Interactive II
0.4 wt% Ald-F(F)F	0.5 wt% Agarose	Orthogonal
0.3 wt% Lys-cycC_5	0.5 wt% Agarose	Interactive II
0.6 wt% APmoc-F(CF_3)F	0.5 wt% Agarose	Orthogonal*
0.4 wt% BPmoc-F_3	0.5 wt% Agarose	Interactive II

Solvent: 100 mM MES pH 7.0 [except for **DBS-COOH** and **APmoc-F(CF_3)F**], H_2O (for **DBS-COOH**).

* previously reported[8] (solvent: 100 mM HEPES, pH 8.0)

Supplementary Table 3. Summary of quantification for network classification (*n* = 3)

The data present the mean ± s.d.

x/<br>0.5 wt% Agarose composite	PCC	Agarose domain size (μm^2)	Agarose Pore size (μm^2)	Standard deviation (*P* value vs agarose)
0.1 wt% **BPmoc-F$_3$**	0.08 ± 0.10	0.244 ± 0.003	0.47 ± 0.06	(1.365 ± 0.007)×10^4 (*ns*)
0.4 wt% **Ald-F(F)F**	-0.01 ± 0.02	0.251 ± 0.008	0.52 ± 0.03	(1.382 ± 0.007)×10^4 (*ns*)
0.3 wt% **GalNAc-MecycC$_6$**	0.38 ± 0.12	0.241 ± 0.006	0.358 ± 0.017	(1.241 ± 0.012)×10^4 ($P < 0.001$)
0.4 wt% **Phos-MecycC$_5$**	0.01 ± 0.02	0.211 ± 0.006	0.32 ± 0.03	(1.264 ± 0.012)×10^4 ($P < 0.001$)
0.4 wt% **NPmoc-F(F)F**	0.09 ± 0.06	0.19 ± 0.02	0.24 ± 0.02	(1.256 ± 0.009)×10^4 ($P < 0.001$)
0.3 wt% **Lys-cycC$_5$**	0.14 ± 0.03	0.222 ± 0.006	0.35 ± 0.03	(1.316 ± 0.007)×10^4 ($P < 0.001$)
0.4 wt% **BPmoc-F$_3$**	0.04 ± 0.13	0.196 ± 0.003	0.228 ± 0.006	(1.2868 ± 0.007)×10^4 ($P < 0.001$)
0.2 wt% **DBS-COOH**	0.23 ± 0.01	0.245 ± 0.003	0.50 ± 0.12	(1.37 ± 0.03)×10^4 (*ns*)
(Single-component Agarose)	-	0.27 ± 0.02	0.51 ± 0.03	(1.373 ± 0.010)×10^4 (control)

Supplementary Table 4. The storage moduli, loss moduli, tan *δ*, and enhancement factor of each hydrogel

	G′ / Pa	*G″* / Pa	tan *δ*	Enhancement factor
0.5 wt% Agarose	738.9	52.0	0.07	-
0.5 wt% Agarose (+Glucono-δ-lactone)	309.3	23.9	0.08	-
0.15 wt% BPmoc-F$_3$	374.9	38.8	0.10	-
0.2 wt% GalNAc-cycC$_6$	464.6	107.2	0.23	-
0.4 wt% Phos-MecycC$_5$	0.3	0.1	0.37	-
0.2 wt% DBS-COOH (+Glucono-δ-lactone)	371.8	40.0	0.11	-
0.15 wt% BPmoc-F$_3$/0.5 wt% Agarose	1719.0	292.5	0.17	1.54
0.2 wt% GalNAc-cycC$_6$/0.5 wt% Agarose	6448.0	834.8	0.13	5.36
0.4 wt% Phos-MecycC$_5$/0.5 wt% Agarose	1748.9	161.9	0.09	2.37
0.2 wt% DBS-COOH/0.5 wt% Agarose (+Glucono-δ-lactone)	1239.5	105.1	0.08	1.82

Supplementary Table 5. The yield strain of each hydrogel

	Yield strain / %
0.5 wt% Agarose	52.1
0.5 wt% Agarose (+Glucono-δ-lactone)	102.8
0.15 wt% BPmoc-F_3	13.4
0.2 wt% GalNAc-cycC_6	21.7
0.4 wt% Phos-MecycC_5	420.0
0.2 wt% DBS-COOH (+Glucono-δ-lactone)	51.1
0.15 wt% BPmoc-F_3/0.5 wt% Agarose	42.0
0.2 wt% GalNAc-cycC_6/0.5 wt% Agarose	11.6
0.4 wt% Phos-MecycC_5/0.5 wt% Agarose	82.1
0.2 wt% DBS-COOH/0.5 wt% Agarose (+Glucono-δ-lactone)	52.1

Supplementary references

1. Ikeda, M. *et al.* Installing logic-gate responses to a variety of biological substances in supramolecular hydrogel-enzyme hybrids. *Nat. Chem.* **6**, 511–518 (2014).
2. Shigemitsu, H. *et al.* Preparation of supramolecular hydrogel–enzyme hybrids exhibiting biomolecule-responsive gel degradation. *Nat. Protoc.* **11**, 1744–1756 (2016)
3. Kubota, R. *et al.* Control of seed formation allows two distinct self-sorting patterns of supramolecular nanofibers. *Nat. Commun.* **11**, 4100 (2020).
4. Matsumoto, S. *et al.* Photo gel–sol/sol–gel transition and its patterning of a supramolecular hydrogel as stimuli-responsive biomaterials. *Chem. Eur. J.* **14**, 3977–3986 (2008).
5. Komatsu, H., Ikeda, M. & Hamachi, I. Mechanical reinforcement of supramolecular hydrogel through incorporation of multiple noncovalent interactions. *Chem. Lett.* **40**, 198–200 (2011).
6. Kubota, R. *et al.* Imaging-based study on control factors over self-sorting of supramolecular nanofibers formed from peptide- and lipid-Type hydrogelators. *Bioconjugate Chem.* **29**, 2058–2067 (2018).
7. Cornwell, D. J., Okesola, B. O. & Smith, D. K. Hybrid polymer and low molecular weight gels – dynamic two-component soft materials with both responsive and robust nanoscale networks. *Soft Matter* **9**, 8730–8736 (2013).
8. Shigemitsu, H. *et al.* Protein-responsive protein release of supramolecular/polymer hydrogel composite integrating enzyme activation systems. *Nat. Commun.* **11**, 3859 (2020).
9. Onogi, S. *et al. In situ* real-time imaging of self-sorted supramolecular nanofibres. *Nat. Chem.* **8**, 743–752 (2016).
10. Nakamura, K. *et al.* Phototriggered spatially controlled out-of-equilibrium patterns of peptide nanofibers in a self-sorting double network hydrogel. *J. Am. Chem. Soc.* **143**, 19532–19541 (2021).
11. Dunn, K. W., Kamocka, M. M. & McDonald, J. H. A practical guide to evaluating

colocalization in biological microscopy. *Am. J. Physiol. Cell Physiol.* **300**, C723–C742 (2011).

12. Schindelin, J. *et al.* Fiji: an open-souce platform for biological-image analysis. *Nat. Methods* **9**, 676–682 (2012).
13. Hoop, K. A., Kennedy, D. C., Mishki, T., Lopinski, G. P., Pezacki, J. P. Silicon and silicon oxide surface modification using thiamine-catalyzed benzoin condensations. Can. J. Chem. 90, 262–270 (2012).
14. Sai, H. *et al.* Imaging supramolecular morphogenesis with confocal laser scanning microscopy at elevated temperatures. *Nano Lett.* **20**, 4234–4241 (2020).
