# Supplementary material for: Four distinct network patterns of supramolecular/polymer composite hydrogels controlled by formation kinetics and interfiber interactions
Source: Nat Commun. 2023 Mar 27;14:1696. doi: 10.1038/s41467-023-37412-0 (PMC10042874; doi:10.1038/s41467-023-37412-0)
Supplement: Supplementary file 3 — Description of Additional Supplementary Files [file 41467_2023_37412_MOESM3_ESM.pdf]

Description of Additional Supplementary Files

File Name: Supplementary Movie 1
Description: Real-time CLSM imaging of the formation of the BPmoc-F_3/Alx488-Agarose composite hydrogel. Conditions: [BPmoc-F_3] = 0.1 wt% (1.6 mM), [TMR-Gua] = 14 μM, [Alx488- Agarose] = 0.5 wt%, solvent: 100 mM MES pH 7.0, scale bar: 5 μm. Elapsed time was displayed as mm:ss:fff.

File Name: Supplementary Movie 2
Description: Real-time CLSM imaging of the formation of the Phos-MecycC$_5$/Alx488- Agarose composite hydrogel. Conditions: [Phos-MecycC$_5$] = 0.4 wt% (6.5 mM), [Alx546-cycC$_6$] = 4 μM, [Alx488-Agarose] = 0.5 wt%, solvent: 100 mM MES pH 7.0, scale bar: 5 μm. Elapsed time was displayed as mm:ss:fff.

File Name: Supplementary Movie 3
Description: Real-time CLSM imaging of the formation of the GalNAc-cycC6 /Alx488- Agarose composite hydrogel. Conditions: [GalNAc-cycC$_6$] = 0.3 wt% (4.6 mM), [Alx546-cycC$_6$] = 4 μM, [Alx488-Agarose] = 0.5 wt%, solvent: 100 mM MES pH 7.0, scale bar: 5 μm. Elapsed time was displayed as mm:ss:fff.

File Name: Supplementary Movie 4
Description: Real-time CLSM imaging of the formation of the DBS-COOH/Alx488-Agarose composite hydrogel. Conditions: [DBS-COOH] = 0.2 wt% (4.5 mM), [TMR-Gua] = 14 μM, [Alx488- Agarose] = 0.5 wt%, [glucono-δ- lactone] = 44.9 mM, solvent: water, scale bar: 5 μm. Elapsed time was displayed as mm:ss:fff.

File Name: Supplementary Movie 5
Description: Real-time CLSM imaging of the formation of the BPmoc-F_3/Alx488-Agarose composite hydrogel with a pH decrease protocol. Conditions: [BPmoc-F_3] = 0.2 wt% (3,2 mM), [TMR-Gua] = 14 μM, [Alx488- Agarose] = 0.5 wt%, [glucono-δlactone] = 44.9 mM, solvent: water, scale bar: 5 μm. Elapsed time was displayed as mm:ss:fff.
